# Loss of T cell tolerance in the skin following immunopathology is linked to failed restoration of the dermal niche by recruited macrophages

**DOI:** 10.1016/j.celrep.2022.110819

**Published:** 2022-05-17

**Authors:** Heather C. West, James Davies, Stephen Henderson, Oluyori K. Adegun, Sophie Ward, Ivana R. Ferrer, Chanidapa A. Tye, Andres F. Vallejo, Laura Jardine, Matthew Collin, Marta E. Polak, Clare L. Bennett

**Affiliations:** 1 Department of Haematology, University College London (UCL) Cancer Institute, London WC1E 6DD, UK; 2 Institute for Immunity and Transplantation, Division of Infection and Immunity, University College London, London NW3 2PF, UK; 3 Bill Lyons Informatics Centre, Cancer Institute, University College London, London WC1E 6DD, UK; 4 Department of Cellular Pathology, University College London Hospitals NHS Foundation Trust, London, UK; 5 Clinical and Experimental Sciences (Sir Henry Wellcome Laboratories, Faculty of Medicine) and Institute for Life Sciences, University of Southampton, Southampton, UK; 6 Biosciences Institute, Newcastle University, Newcastle Upon Tyne, UK; 7 Newcastle University Translational and Clinical Research Institute and NIHR Newcastle Biomedical Research Centre, Newcastle University, Newcastle Upon Tyne, UK

**Keywords:** skin, monocytes, macrophages, dermis, graft-versus-host disease, disease niche, regulatory T cells, tolerance, contact hypersensitivity

## Abstract

T cell pathology in the skin leads to monocyte influx, but we have little understanding of the fate of recruited cells within the diseased niche, or the long-term impact on cutaneous immune homeostasis. By combining a murine model of acute graft-versus-host disease (aGVHD) with analysis of patient samples, we demonstrate that pathology initiates dermis-specific macrophage differentiation and show that aGVHD-primed macrophages continue to dominate the dermal compartment at the relative expense of quiescent MHCII^int^ cells. Exposure of the altered dermal niche to topical haptens after disease resolution results in hyper-activation of regulatory T cells (Treg), but local breakdown in tolerance. Disease-imprinted macrophages express increased IL-1β and are predicted to elicit altered TNF superfamily interactions with cutaneous Treg, and we demonstrate the direct loss of T cell regulation within the resolved skin. Thus, T cell pathology leaves an immunological scar in the skin marked by failure to re-set immune homeostasis.

## Highlights

• Skin immune pathology drives dermis-specific monocyte differentiation
• GVHD-primed macrophages dominate the dermis after disease
• T cell tolerance to topical haptens fails despite recovery from GVHD
• Tregs are dysfunctional within the altered dermal niche

## Graphical abstract

**Figure:**
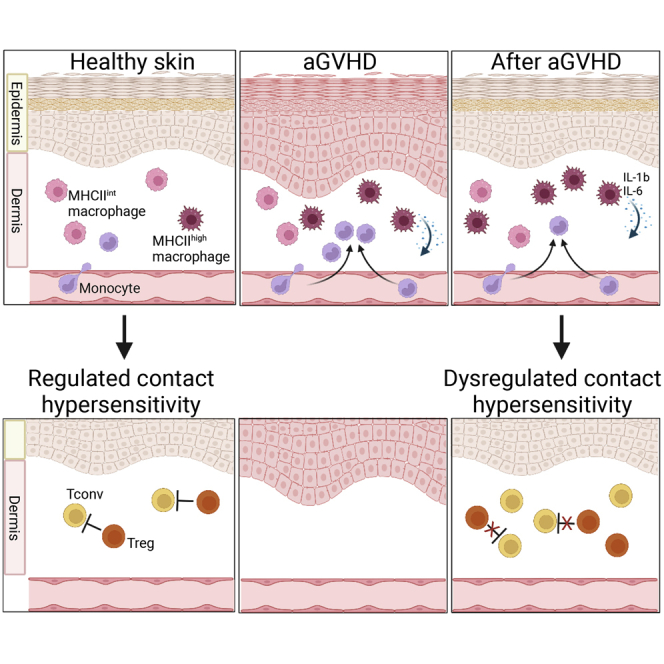

## Teaser

The impact of T cell pathology on cutaneous immunity is not well understood. Here, West et al. combine a model of graft-versus-host-disease with patient data to reveal prolonged dysregulation of dermal macrophage differentiation, and breakdown in Treg function, leading to a failure to re-set the skin immune balance after disease.

## Introduction

Immune homeostasis at barrier tissues is laid down early in life but must be actively maintained to limit activation of potentially pathogenic effector T cells in the absence of infection (Guttman-Yassky et al., 2019). Acute graft-versus-host disease (aGVHD) after hematopoietic stem cell transplant is a major cause of morbidity and mortality, severely limiting the success of treatment (Zeiser and Blazar, 2017). The skin is a primary target organ in aGVHD, during which donor T cell-mediated destruction of allogeneic patient cells leads to significant remodeling of the cutaneous immune network (Haniffa et al., 2009; Mielcarek et al., 2014; Wang et al., 2014). Our recent studies have suggested the importance of local skin-specific mononuclear phagocytes in the pathology of aGVHD (Jardine et al., 2020; Santos et al., 2018). But we understand little about how the diseased myeloid niche impacts on recovery of immune homeostasis and local regulation of T cell function in the skin.

Macrophages dominate the dermal immune compartment in the steady state where, in addition to core functions in innate immune protection, they are essential for wound repair and restoration of tissue homeostasis after damage (Kabashima et al., 2019; Kolter et al., 2019). Murine studies suggest that dermal macrophages are largely short-lived, and frequently replaced by circulating Ly6C^+^ monocytes (Tamoutounour et al., 2013), thus resembling their counterparts within the gut lamina propria (Bain et al., 2013). But recently studies have highlighted heterogeneity within the dermis, suggesting that some specialized dermal macrophages are longer-lived (Baranska et al., 2018; Kolter et al., 2019), in agreement with data from the human dermis (Haniffa et al., 2009). In particular, monocyte-specific fate-mapping studies have highlighted the differential turnover within two major subsets of dermal macrophages (Liu et al., 2019): MHCII^high^ macrophages are constitutively maintained by circulating Ly6C^+^ monocytes in the adult (Liu et al., 2019), and are likely to be primed for pro-inflammatory responses (Guilliams and Svedberg, 2021; Louwe et al., 2021); while the transition to MHC^int^ macrophages is associated with resolution of disease, tissue repair, and a return to homeostasis (Chakarov et al., 2019; Kolter et al., 2019).

Within the epidermis, aGVHD leads to replacement of the resident Langerhans cell (LC) network after T cell-mediated destruction of host cells (Ferrer et al., 2019; Mielcarek et al., 2014). But key questions remain about how recruitment of monocytes into the diseased niche will impact on the differentiation and maintenance of the quiescent macrophages in the dermal compartment. Donor T cells drive differentiation of pathogenic monocytes in GVHD (Tugues et al., 2018), and differentiation of a distinct population of dermal monocyte-derived macrophages in patient lesions (Jardine et al., 2020), while inflammatory monocytes contribute to fibrosis in chronic GVHD-like disease (Haub et al., 2019). Together these studies highlight the importance of patient donor monocyte-derived cells for disease, but the long-term impact of the pathogenic monocyte response on the balance of immunity by other immune cells in the skin could not be addressed in patient studies.

Crosstalk between mononuclear phagocytes and T cells is required to maintain immune balance at barrier sites, and chronic disease is frequently characterized by parallel dysfunction within myeloid and lymphoid compartments (Castro-Dopico et al., 2020). Dysregulation of mononuclear phagocytes impacts T cell homeostasis (Mortha et al., 2014; Wehrens et al., 2013), and auto-inflammation in the skin and other organs is frequently associated with the hyper-activation, but loss of function of regulatory T cells (Treg) (Pesenacker et al., 2015). Within the skin, interaction between myeloid cells and T cells controls protective skin immunity (Naik et al., 2015) and adaptation to injury (Harrison et al., 2019). Conversely, loss of immune homeostasis is frequently associated with hypersensitivity to topical antigens and the development of allergic dermatitis, and an absence of Treg-mediated restraint is sufficient to induce spontaneous inflammatory disease (Hartwig et al., 2018).

Here, we examined how pathogenic T cells re-shaped the dermal monocyte-macrophage compartment during and after aGVHD and questioned how disease impacted on the long-term balance of immune regulation by T cells in the skin. We demonstrate that T cell pathology leads to the expansion of a distinct population of inflammatory differentiated monocytes in both murine and human aGHVD, and psoriatic skin. Moreover, continued recruitment of inflammatory monocytes beyond resolution of disease appears to drive a chronic tissue state in which MHCII^high^ inflammatory macrophages predominate, at the expense of quiescent resident cells. By testing the induction of tolerance to topical hapten, we demonstrated an absence of T cell regulation, and development of exaggerated contact dermatitis when topical antigen was applied to the skin after the resolution of aGVHD. Cell-cell interaction analysis of human cells suggested dysregulation of TNF family super-member-mediated crosstalk between inflammatory monocytes and Treg in the context of T cell pathology, which was functionally demonstrated by the loss of suppressive function in dermal Treg. Our work therefore highlights the concept of long-term immunological scarring within dermis in which the parallel breakdown in resident macrophage differentiation and Treg dysfunction precludes restoration of an immune equilibrium.

## Results

### Discrete compartment-specific differentiation of monocytes in aGVHD skin

**Figure 1:**
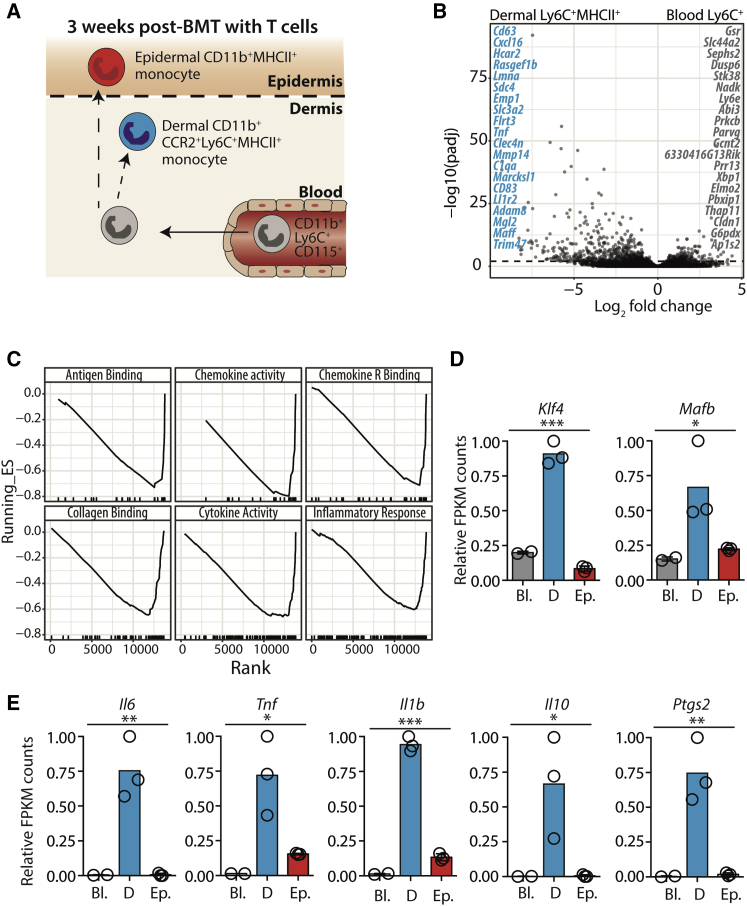
Discrete compartment-specific differentiation of monocytes in aGVHD skin Analysis of RNA-seq data from monocytes sorted from the blood (CD11b^+^Ly6C^high^CD115^+^; n = 2) and monocyte-derived cells in the dermis (CD24^neg^CCR2^+^Ly6C^+^MHCII^+^; n = 3) and epidermis (CD11b^+^MHCII^+^CD24^low^Langerin^neg^; n = 3) 3 weeks post-BMT with T cells (GSE130257). (A) Schematic showing sorted cell populations. (B) Volcano plot highlighting the top genes upregulated in dermal versus blood monocytic cells. Genes with Log2(FC) > ±2 and FDR adjusted p value less than 0.01 were considered significant. (C) Graphical output from gene set enrichment analysis showing enriched gene ontogeny sets in dermal monocytes compared with blood monocytes. (D and E) Graphs show DESeq counts normalized to the maximum value for different cytokines (D) or transcription factors (E). Significance was calculated with one-way ANOVA. ^∗^p < 0.05, ^∗∗^p < 0.01, ^∗∗∗^p < 0.001. Bl., blood monocytes; D, dermal monocyte-derived cells; Ep., epidermal monocyte-derived cells.

Adoptive transfer of male minor histo-compatibility antigen-reactive Matahari (Mh) T cells with bone marrow (BM) transplantation (BMT) results in Mh T cell priming and recruitment to target organs, where cytotoxicity leads to a sub-lethal aGVHD that is evident from 2 to 3 weeks post-transplant but resolves over time (Santos et al., 2018; Toubai et al., 2012). At 6 weeks post-transplant mice have regained weight without development of overt chronic GVHD, as measured by the thymic T cell phenotype, and lack of pathology in salivary glands (Figure S1). Within the skin, pathogenic Mh T cells create an altered disease niche in the epidermis that is highly accessible to circulating monocytes, resulting in the remodelling of the LC network by monocyte-derived cells (Ferrer et al., 2019). To define the impact of aGVHD on the dermal and epidermal tissue environments in this model, we conducted a proteomic screen of secreted factors from total epidermal or dermal cells isolated from control mice that had received BMT alone, or mice that had received BMT with Mh T cells 3 weeks previously. Within the epidermis we observed a broad upregulation of chemokines and growth factors, suggesting activation of cell recruitment and tissue repair mechanisms (Figure S2A). By comparison, the changes to the dermal secretome were more limited (Figure S2B) but dominated by the upregulation of inflammatory proteins (IL-6, GM-CSF), chemokines (CXCL12, CCL5), and proteins associated with fibrosis (PDGF-BB), mirroring the skin of patients with GVHD-like disease (Taylor et al., 2018). To understand whether these differences were reflected in the immediate fate of monocytes recruited into the two sites we mined our published gene expression dataset of myeloid cells isolated from the skin 3 weeks after BMT with T cells (Ferrer et al., 2019). This dataset allowed us to directly compare the transcriptome of blood Ly6C^+^ monocytes with differentiated MHCII^+^ monocytes within the inflammatory dermal or epidermal aGVHD environments (Figure 1A). Transition from blood Ly6C^+^ cells to dermal Ly6C^+^MHCII^+^ monocytes was associated with increased expression of genes associated with propagation of the inflammatory response due to interactions with other immune cells (*cxcl16*, *tnf*, *cd83*, *il1r2*, *mgl2*, *maff*) and the extracellular environment (*cd63*, *sdc4*, *flrt3*, *clec4n*, *mmp14*, *adam8*), and with vasodilation (*har2*) (Figure 1B). These data were consistent with the concept of tissue monocyte-derived cells as coordinators of the local leukocyte and tissue environment; indeed, gene set enrichment analysis suggested that differentiation in the dermis equipped monocytes with the potential to modulate immune cells and extracellular matrix in the local tissue environment (Figure 1C). In contrast, despite the inflammatory environment, entry into the epidermis did not induce a clear inflammatory transcriptional response, but activated processes of cell division (Figures S2C and S2D) (Ferrer et al., 2019). Direct comparison of the expression of specific genes within monocyte-derived cells isolated from the two sites reinforced the clear divergence in monocyte-derived cell fate, as illustrated by dermal expression the transcription factors *Klf4* and *Mafb*, both associated with differentiation of monocyte-derived dendritic cells (DCs) (Goudot et al., 2017; Jurkin et al., 2017) (Figure 1D). Likewise, the term “inflammatory response,” which included pro-inflammatory genes and those classically associated with control of immunity, such as via production of PGE_2_ (*Ptsg2*), was selectively enriched in dermal cells compared with those in the epidermis (Table S1) (Figure 1E).

Thus, initiation of cutaneous aGVHD leads to the distinct compartmentalized differentiation of recruited monocytes with the potential to condition the tissue environment.

### T cell pathologies elicit a common program of macrophage differentiation in human skin

**Figure 2:**
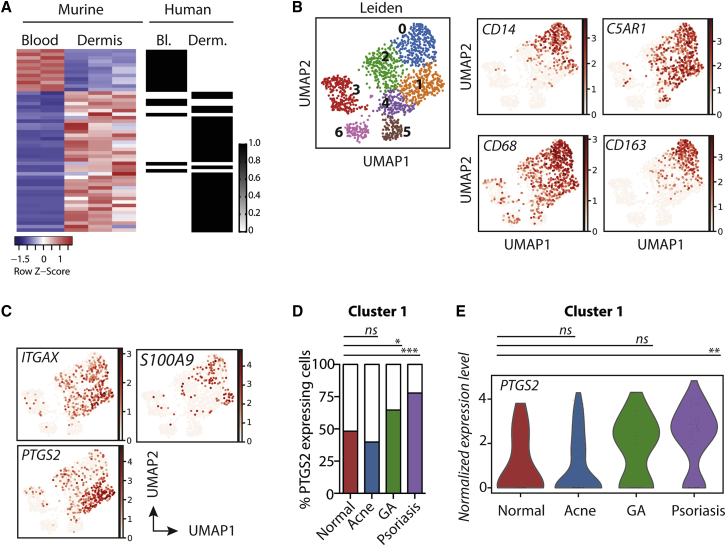
T cell pathologies elicit a common program of monocyte differentiation in human skin (A) RNA-seq data from murine aGVHD blood (CD11b^+^Ly6C^high^CD115^+^; n = 2) and dermal (CD24^neg^CCR2^+^Ly6C^+^MHCII^+^; n = 3) monocytes was compared with NanoString data from human aGVHD blood (CD14^+^, n = 5) and dermal (CD14^+^CD11c^+^, n = 3) monocytes. Heatmaps display comparison of 52 differentially expressed genes upregulated in both murine aGVHD and human aGVHD dermal monocytes. (B–D) Analysis of human skin single-cell data from Hughes et al. (GSE150672), comprising whole skin from healthy, psoriasis, acne, granulomatous annuloma (GA), and alopecia patients. (B) Left: UMAP displaying DC/monocyte/macrophage clusters after exclusion of LCs. Right: marker plots show monocyte and macrophage-associated genes co-localized in clusters 0 and 1. (C) Marker plots show *ITGAX*, *S100A9*, and *PTGS2* gene expression, used to identify the CD11c^+^CD14^+^ monocyte sub-cluster. (D) Bar graph shows the frequency of cells in cluster 1 that express (colored bars) or do not express (white bars) *PTGS2* in healthy or diseased skin. Normal versus GA p = 0.036, versus psoriasis p < 0.0001. (E) Violin plots show normalized expression of *PTGS2* in cluster 1 monocytes from normal, acne, GA and psoriasis samples. Normal versus psoriasis p = 0.01231816 at Log FC = 2.2930138 (t test). ^∗^p < 0.05, ^∗∗^p < 0.01, ^∗∗∗^p < 0.001; ns, not significant.

CD11c^+^CD14^+^ monocyte-derived macrophages dominate the immune cell infiltrate in patient aGVHD lesions (Jardine et al., 2020). To determine the clinical relevance of murine aGVHD Ly6C^+^MHCII^+^ monocyte-derived cells, we identified the 52 genes that were mutually differentially regulated when comparing blood with dermal monocyte/macrophage populations in both our murine model and aGVHD patients (Jardine et al., 2020) (Table S2) and compared expression of these genes between species. Figure 2A illustrates the significant overlap between the human and murine gene profiles (hypergeometric test: human and mouse blood monocytes, p = 1.1 × 10^−43^; human skin CD11c^+^CD14^+^ monocyte-derived cells and murine Ly6C^+^MHCII^+^ monocytes, p = 2.0 × 10^−102^), suggesting that the aGVHD environment generates homologous populations of phenotypically distinct monocytes in both murine and patient skin. To determine whether this program of monocyte differentiation was unique to the aGVHD lesions or elicited by other T cell-mediated skin diseases, we investigated a recently published single-cell dataset from healthy and diseased skin (Hughes et al., 2020). Data were extracted and combined knowledge of known cell-type-defining genes with automatic cell reference database annotation tools (Single R) used to define clusters of cells that were likely to be total skin mononuclear phagocyte populations, namely LCs, dermal DCs, macrophages, and monocytes (Figures S3A–S3E). To focus on dermal cells, we excluded LCs from this analysis and focused on the remaining myeloid populations (Figure 2B). This analysis clearly identified clusters 0 and 1 as monocyte/macrophages and showed that all six DC populations that have been identified in the blood (Bourdely et al., 2020; Villani et al., 2017) can be detected in the dermis (Figure S3F and see Table S3 for gene lists). Focusing on cluster 1 demonstrated a sub-population of *ITGAX*^*+*^*CD14*^*+*^*S100A9*^*+*^*PTGS2*^*+*^ cells (Figures 2B and 2C), suggesting that emergence of CD11c^+^CD14^+^ macrophages was not limited to cutaneous aGVHD. Using *PTGS2* as a defining marker of the monocyte-derived macrophages in cluster 1, we subsequently tested the hypothesis that emergence of these cells depends on T cell pathology in the skin. Figures 2D and 2E demonstrate that, indeed, granulomatous annuloma and psoriatic skin, both driven by aberrant activation of pathogenic T cells, but not acne skin, contained a higher frequency of *PTSG2*-expressing macrophages than in healthy skin, and that these cells displayed, on average, higher expression of the *PTSG2* gene.

We conclude that the dermal inflammatory monocyte-derived cells defined in our murine aGVHD model mirror the pathogenic CD11c^+^CD14^+^ macrophages within patient aGVHD lesions; and that T cell pathologies elicit a common program of differentiation and accumulation of immunoregulatory monocytes in the skin.

### Inflammatory monocyte-derived cells dominate the dermal macrophage compartment after aGVHD

**Figure 3:**
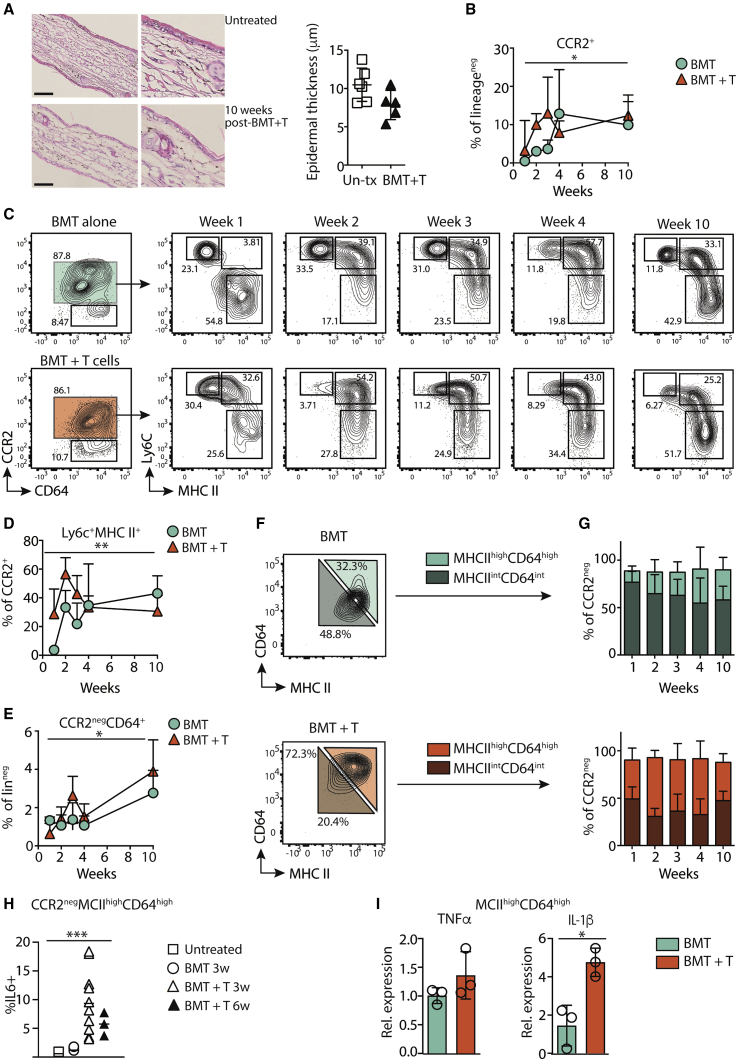
Recruited monocytes fail to re-set the dermal macrophage compartment after aGVHD (A) Left: representative images show H&E stains at 20× magnification, and digital zoomed in sections, of ear histology from age-matched untreated mice or recipients of BMT with T cells 10 weeks post-transplant. Scale bars, 100 μm. Right: summary graph showing epidermal thickness (mean ± SD) across sections. Symbols show individual mice, untreated n = 6, BMT + T n = 5. (B–G) Male recipients received female bone marrow alone (BMT; circles) or with T cells (BMT + T; triangles). The frequency of dermal myeloid populations was measured over time post-transplant. (B) Graph shows the frequency ± SD of CCR2^+^ cells of lineage^neg^ dermal cells. (C) Representative contour plots show sub-populations of pre-gated single, lineage^neg^, CD11b^+^CCR2^+^cells within the dermis at different time point post-transplant. (D) Summary line graph showing the frequency ± SD of Ly6C^+^MHCII^+^ cells within CCR2^+^ cells over time. (E) Summary line graph showing the frequency ±SD of CCR2^neg^CD64^+^ cells dermal cells over time. (F) Representative contour plots to show gating of CCR2^neg^ cell populations for (G). (G) *S*ummary stacked bar graphs show the frequency ± SD of MHCII^int^CD64^int^ (darker bars) or MHCII^high^CD64^high^ (lighter bars) within dermal CCR2^neg^ cells at different time points post-transplant. Data for (B)–(G) are pooled from two independent experiments for each time point (BMT n = 5–7, BMT + T n = 7–8) and analyzed by two-way ANOVA; MHCII^low^ cells or MHCII^high^ cells BMT versus BMT+ T p < 0.0001. (H) Summary graph showing the frequency of IL-6^+^ cells within CCR2^neg^MHCII^high^CD64^high^ cells. Symbols are individual mice, and the line shows the mean: untreated n = 4; BMT 3 weeks (3w) n = 6, BMT + T 3 weeks (3w) n = 11 and 6 weeks (6w) n = 3. Data are pooled from two independent experiments and analyzed by one-way ANOVA. (I) Bar graphs show mean ± SD gene expression relative to β-actin from sorted CCR2^neg^MHCII^high^CD64^high^macrophages isolated from BMT or BMT + T recipients 7 weeks post-transplant. Symbols are pooled experimental replicates from two independent experiments, n = 3 per group. Data were analyzed using a Mann-Whitney test. ^∗^p < 0.05, ^∗∗^p < 0.01, ^∗∗∗^p < 0.001.

Having demonstrated the conserved expansion of inflammatory monocytes across human and murine cutaneous T cell pathologies, we asked how accumulation of inflammation-imprinted (Guilliams and Svedberg, 2021) monocyte-derived cells in the dermis impacted on maintenance of the local resident macrophage compartment. Thus, we returned to our murine model and tracked the differentiation of Ly6C^+^ monocytes into CCR2^neg^MHCII^low^ dermal macrophages over 10 weeks post-transplant using a gating strategy that encompassed the range of monocyte/macrophage populations in the dermis (Baranska et al., 2018) (Tamoutounour et al., 2013) (Figure S4A). In our model, cutaneous aGVHD is evident histologically in ear skin 3 weeks post-transplant (Santos et al., 2018). By 10 weeks post-transplant, mice appeared healthy, ear skin was microscopically indistinguishable from non-transplanted, age-matched controls (Figure 3A), did not show evidence of overt fibrosis according to staining with Masson’s trichrome (Figure S4B), and epidermal monocytes had differentiated into quiescent steady-state LCs (Ferrer et al., 2019). Irradiation and BMT resulted in the recruitment of donor BM-derived monocytes to the dermis (Figure S4C). Influx of CCR2^+^ monocytes increased the frequency of these cells up to 3 weeks post-BMT with Mh T cells, which then plateaued up to the 10-week final time point (Figure 3B). Recruitment of CCR2^+^ cells was delayed in irradiated controls but reached similar levels as aGVHD mice by 10 weeks post-transplant, consistent with a low-grade GVH response in these mice (Figure 3B). Within dermal CCR2^+^ cells, inflammatory Ly6C^+^MHCII^+^ monocytes differentiated more rapidly, and to a greater extent in the context of aGVHD (Figure 3C) and peaked at the height of Mh T cell accumulation at this site (Figures 3D and S4D). Focusing on the emergence of CCR2^neg^CD64^+^ differentiated dermal macrophages demonstrated that this compartment was expanded over time post-transplant, ultimately resulting in a similar frequency of CCR2^neg^CD64^+^ cells in mice that had or had not received Mh T cells (Figure 3E). Chimerism analysis of macrophage populations 10 weeks post-transplant was consistent with active contribution of BM-derived donor monocytes to MHCII^high^CD64^high^ macrophages, while a sub-population of long-lived quiescent host-derived MHCII^int^CD64^int^ resident cells persisted in the dermis, as described by others (Haniffa et al., 2009; Kolter et al., 2019; Liu et al., 2019) (Figure S4E). Despite the apparent comparable repopulation of the dermal niche by CCR2^neg^ macrophages, we observed that MHCII^high^CD64^high^ cells remained more prevalent within the dermal macrophage compartment up to 10 weeks post-transplant with Mh T cells (MHCII^high^CD64^high^ cells BMT versus BMT + T two-way ANOVA p < 0.001), resulting in a reduction in the relative frequency of MHCII^int^CD64^int^ resident macrophages compared with BMT only controls (Figures 3F and 3G). *In vivo* production of intracellular IL-6 without restimulation of dermal cells discriminated between CD11b^+^ myeloid cells isolated from aGVHD or control dermis (Figure S4F), and MHCII^high^CD64^high^ macrophages remained primed for the inflammatory response 6 weeks post-transplant (Figure 3H). To determine how the impaired restoration of the baseline macrophage compartment would alter the dermal tissue environment following disease, we analyzed the functional differences between CCR2^neg^ macrophage populations in mice that had received BMT or BMT with T cells. Direct *ex vivo* TNF-α production demonstrated that MHCII^high^CD64^high^ macrophages were more pro-inflammatory than MHCII^int^CD64^int^ cells (Figure S4G), consistent with the association between downregulation of MHCII and differentiation to a more quiescent state (Liu et al., 2019; Louwe et al., 2021); however, this appeared to be a constitutive response to BMT that was not driven by aGVHD (Figure 3I). By contrast, MHCII^high^CD64^high^ macrophages from mice that had experienced aGVHD continued to produce higher levels of IL-1β (Figure 3I), suggesting prolonged functional changes to the dermal macrophage compartment in these mice.

Therefore, aGVHD results in a dysregulated dermal niche in which recruited monocytes appear unable to restore the quiescent MHCII^int^ resident macrophage compartment. Continued differentiation of inflammatory macrophages reinforces this chronic inflammatory environment beyond resolution of T cell-mediated disease.

### Recovery from aGVHD is associated with long-term loss of T cell regulation in the skin

**Figure 4:**
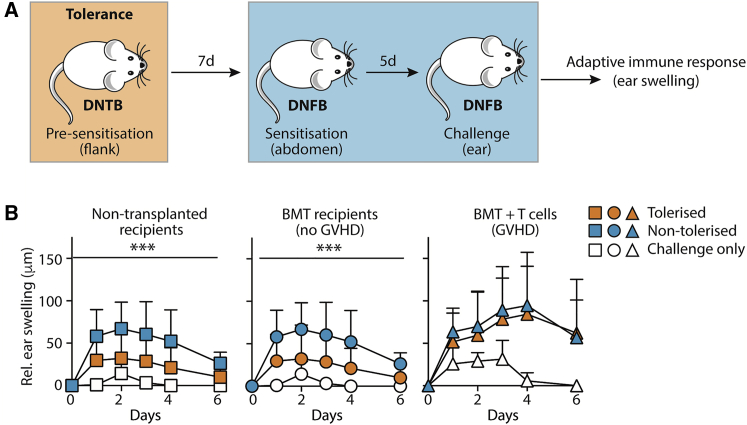
Recovery from aGVHD is associated with long-term loss of T cell regulation in the skin (A) Schematic showing the experimental protocol to elicit tolerance (pre-sensitization) or an active immune response (no pre-sensitization) in untreated mice, or those that had received BMT, with or without T cells, 10 weeks before. (B) Line graphs show the relative ear swelling (±SD) in the challenged right ear compared with the non-challenged left ear in non-transplanted or BMT controls or mice that had received BMT with T cells. Data are pooled from two independent experiments, which contained all recipients and treatments, n = 9–12 per group; two-way ANOVA, ^∗∗∗^p < 0.001.

Given the fine balance between tissue myeloid and lymphoid cells in orchestrating immune homeostasis at barrier sites (Mortha et al., 2014; Whibley et al., 2019), we postulated that a functional impact of the skewed dermal macrophage compartment would be loss of T cell-mediated regulation of homeostasis. Cutaneous immune regulation may be modeled by the induction of tolerance to topical haptens, in which Treg control local effector T cell responses within dermal macrophage niches (Kabashima et al., 2019; Natsuaki et al., 2014; Vocanson et al., 2010). Thus, 10 weeks post-BMT with or without T cells, and after recovery from aGVHD in the T cell group, mice were sensitized and challenged with topical 2,4-dinitrofluorobenzene (DNFB) to elicit a classical contact hypersensitivity (CHS) response. Some mice also received a pre-sensitization tolerizing dose of the related hapten 2,4-dinitro-1-thiocyanobenzene (DNTB) (Figure 4A). This tolerizing step results in Treg-dependent suppression of cutaneous effector T cells and consequent reduction in ear swelling (Gomez de Aguero et al., 2012; Vocanson et al., 2010). Topical application of DNFB led to a regulated CHS response in the control BMT and untreated groups, while pre-sensitization with DNTB reduced the magnitude of the ear-swelling response, consistent with the induction of tolerance to DNFB (Figure 4B). By contrast, exposure of skin to topical haptens 10 weeks post-GVHD did not induce tolerance. Instead, we observed a dysregulated CHS response that was both increased in magnitude and duration, and which could not be limited by pre-sensitization with DNTB (Figure 4B).

These data demonstrate a long-term functional impact of cutaneous aGVHD, characterized by a breakdown in the regulation of T cell responses to topical antigen.

### Hyper-activation and expansion of ICOS^+^ Treg in draining lymph nodes after aGVHD

**Figure 5:**
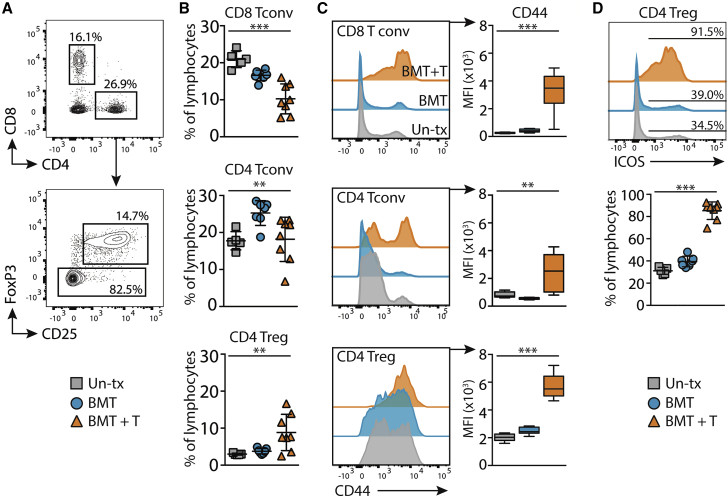
Hyper-activation and expansion of ICOS^+^ Treg in draining LNs after aGVHD Control untreated mice (un-tx), or those that had received BMT with or without T cells at least 6 weeks earlier were pre-sensitized with DNTB and sensitized with DNFB 7 days later. LN T cells were analyzed 5 days post sensitization. (A) Representative contour plots showing gating of T cell populations within a single cell, FSC/SSC lymphocyte gate. (B) Summary graphs show the frequency of gated T cells of total lymphocytes. Symbols represent individual mice with bars showing mean ± SD; squares, untreated; circles, BMT alone; triangles, BMT with T cells. (C) Representative histogram overlays (left), and summary data (right) showing CD44 expression levels (median fluorescent intensity) on gated T cell populations from the different groups. Box and whiskers plots show min to max. (D) Representative histogram overlay (top) and summary graph (bottom) showing the frequency of ICOS^+^ Treg of total lymphocytes. Symbols represent individual mice with bars showing mean ± SD. Data are pooled from two independent experiments: untreated n = 5; BMT only n = 7; BMT + T cells n = 8. Significance was calculated using a one-way ANOVA. ^∗^p < 0.05, ^∗∗^p < 0.01, ^∗∗∗^p < 0.001.

The inability to induce tolerance in mice that had recovered from T cell pathology in the skin suggested aberrant priming of CD4 Treg in draining lymph nodes (LNs). However, analysis of T cell expansion and activation in mice that had received the tolerance protocol revealed a highly specific increase in the relative frequency of CD4 Treg in mice that had received BMT with T cells compared with un-transplanted mice or those that had received BMT alone (Figures 5A and 5B). This was associated with relatively stable frequencies of CD4 conventional T cells (Tconv) across the groups, but a marked decrease in the frequency of CD8 Tconv in mice that had recovered from aGVHD, suggesting egress of activated T cells out of the LNs (Figures 5A and 5B). Analysis of CD44 expression as a marker of antigen-experienced T cells demonstrated increased CD44 expression on all T cells in the LNs of post-aGVHD mice compared with controls (Figure 5C), suggesting the efficient activation of the effector T cells response in these mice.

High-level expression of the inducible T cell co-stimulator (ICOS) imparts enhanced suppressive function on Treg (Busse et al., 2012; Li and Xiong, 2020), and ICOS^+^ Treg suppress CD8 T cells elicited by topical DNFB (Gomez de Aguero et al., 2012; Vocanson et al., 2010). Phenotyping of LN CD4 Treg in mice that had previously experienced aGVHD demonstrated that the majority of cells expressed high levels of ICOS compared with less than half the LNs Treg in controls that had not suffered from skin disease (Figure 5D).

We conclude that the inability to induce tolerance post-aGVHD was not due to a reduction in the priming of functional Treg, demonstrated by accumulation of hyper-activated ICOS^+^ CD4 Treg in the LNs of these mice.

### Breakdown in local crosstalk between dermal monocyte-derived cells and Treg predicates local Treg dysfunction post-aGVHD

**Figure 6:**
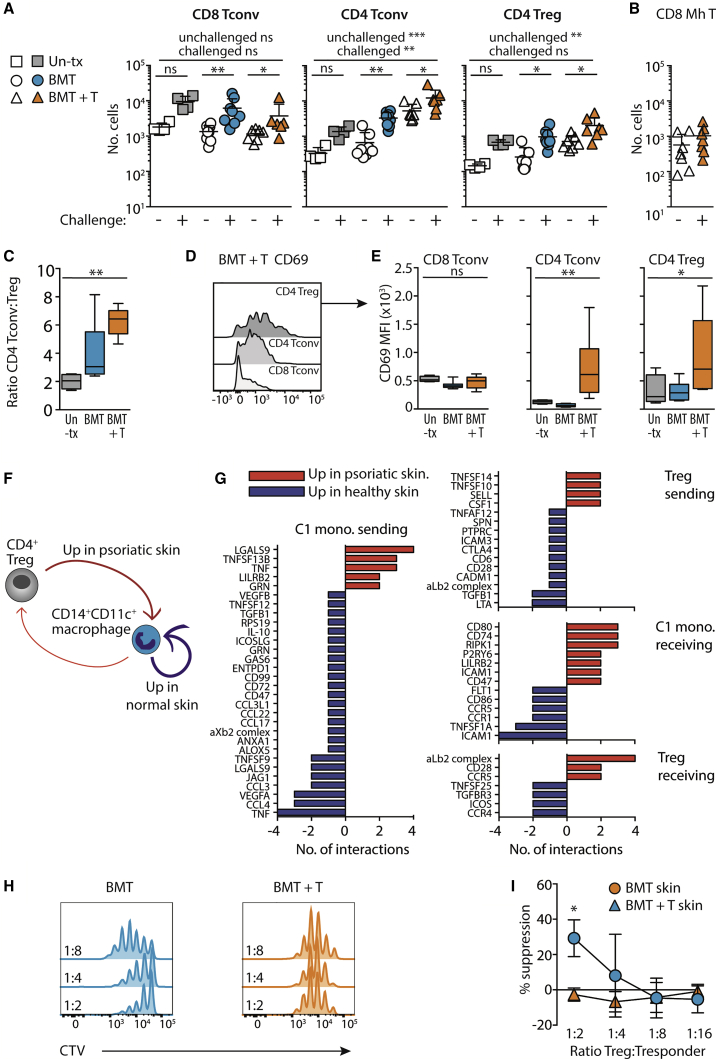
Breakdown in local crosstalk between dermal monocyte-derived cells and Treg predicates local Treg dysfunction post-aGVHD (A–E) Control untreated mice (un-tx), or those that had received BMT with or without T cells 6 weeks earlier, were pre-sensitized with DNTB, then sensitized and challenged with DNFB. Dermal lymphocytes were analyzed 7 days post-challenge. (A) Graphs show the number of CD4 and CD8 Tconv and CD4 Treg in the dermis the unchallenged (−) or challenged (+) ear. Significance between the unchallenged and challenged ear was determined using a paired Wilcoxon test; and differences between transplant groups determined using a one-way ANOVA. (B) Graph showing the number of CD8^+^ Matahari (Mh) T cells in the dermis of the unchallenged (−) or challenged (+) ear. Bars show mean ± SD, data are pooled from two independent experiments. (C) Box and whiskers graph showing the ratio (min to max) between numbers of CD4 Tconv and Treg in the challenged ear. (D) Representative histograms show CD69 expression (median fluorescent intensity) on gated dermal lymphocytes. (E) Summary box and whiskers graphs show the median fluorescent intensity (min to max) of CD69 on gated lymphocytes. Data for (A)–(E) are pooled from two independent experiments: untreated n = 4; BMT only n = 8; BMT + T cells n = 7. Significance was calculated using a one-way ANOVA. (F) Schematic representation of the receptor-ligand analysis cell-cell interaction plot between human CD14^+^CD11c^+^ cluster 1 (C1) monocytes and CD4^+^ Treg. (G) Bar graphs showing the number of interactions received and sent between C1 monocytes and Treg. Red bars indicate interactions that are upregulated in psoriatic skin, and blue bars indicate those unregulated in healthy skin. (H) Representative histograms showing division of gated CD4^+^CD25^low^CD127^high^ responder T cells incubated with CD4^+^CD25^high^CD127^low^ T cells from mice that had received BMT with or without T cells. (I) Summary line graph showing the percentage (±SD) suppression of proliferation compared with responder T cell only controls. Data are pooled from two independent experiments using T cells sorted and pooled from multiple transplanted mice. Significance was calculated with a two-way repeated measures ANOVA, with Sidak’s multiple comparisons test. BMT versus BMT+ T at 1:2 ratio p = 0.03. ^∗^p < 0.05, ^∗∗^p < 0.01, ^∗∗∗^p < 0.001; ns, not significant.

The efficient T cell priming in skin-draining LNs suggested that loss of regulation in the skin was either due to a defect in recruitment of Treg, localized T cell dysfunction, or both. To distinguish between these possibilities, we set out to quantify numbers of cutaneous T cells recruited to the skin using an optimized flow cytometric staining panel that allowed for the sensitivity of the CD8 epitope to tissue digestion enzymes (Autengruber et al., 2012); here dermal Treg were identified as CD4^+^FoxP3^+^ cells, to reflect the heterogenous expression of CD25 in the skin (Ikebuchi et al., 2019) (Figure S5). Untreated mice, or those that had received BMT, with or without T cells, were treated with our tolerance protocol and T cell numbers enumerated 5 days later in the unchallenged and challenged ear skin. We observed increased baseline CD4 Tconv and Treg numbers in the unchallenged skin of mice that had received BMT with T cells compared with controls that had not experienced immune pathology, implying altered T cell homeostasis in these animals prior to challenge (Figure 6A). However, application of topical hapten led to the further specific expansion of dermal T cells above the unchallenged ear in all groups (Figure 6A). The response in the challenged ear was specific to endogenous polyclonal T cells and not to resident monoclonal Mh T cells in mice that had received BMT + T cells (Figure 6B). There was no difference in the numbers of Treg in the dermis between groups, indicating that Treg were recruited to the challenge site in all groups, but that the heightened ratio of Treg to Tconv observed in LNs was not maintained in the skin post-aGVHD. Instead, CD4 Tconv accumulated in significantly higher numbers in the challenged skin of mice that had experienced aGVHD (Figure 6A), resulting in a marked excess of CD4 Tconv compared with Treg within the altered dermal niche (Figure 6C). All CD4 T cells within the post-aGVHD skin were characterized by increased expression of CD69 (Figures 6D and 6E), suggesting local activation within the dermis and consistent with exposure to antigen in the skin (Cibrian and Sanchez-Madrid, 2017; Jaigirdar et al., 2017).

Since Treg accumulated in the dermis, we posited that loss of tolerance post-aGVHD was due to local Treg dysfunction within the altered macrophage environment. Therefore, to determine how inflammatory dermal macrophages might directly impact on Treg function we used an algorithm (CellPhoneDB) to predict receptor-ligand interactions between human cluster 1 (C1) CD11c^+^CD14^+^ macrophages (Figure 2B) and CD4^+^ Treg within healthy and psoriatic skin as a model for T cell pathology. The cell-cell interaction plot represented in Figure 6F, and shown in Figure S6, summarized all predicted interactions between these cell types under both conditions, and demonstrated that, while macrophage-macrophage interactions dominated cell behavior in healthy skin (blue arrow), interactions between Treg and C1 macrophages were enhanced within the psoriatic environment (red arrows) (Figures 6F and S6). As shown in Figure 6G, assessment of the sending and receiving genes from the cell gene interaction analysis (listed in full in Table S4) highlighted the production of TNF superfamily (TNFSF) members within psoriatic skin that were implicated in subverting Treg function (C1 mac. *TNF*) and controlling T cell survival (Treg *TNFSF10*, *TNFSF14*). C1 macrophages in psoriatic skin were also more likely to recruit Treg via CCR5 (C1 mono. *CCL3*/*CCL4* with Treg *CCR5* mean number of receptor-ligand interactions (RL) = +2.25 and +1.19, respectively) and provide signal 2 co-stimulation in the absence of the TNFSF receptor ICOS (C1 mac. *ICOSL* with Treg *ICOS* mean RL = −0.66). Together, these findings suggested that direct and indirect interaction between CD11c^+^CD14^+^ C1 macrophages and Treg in diseased skin was likely to result in loss of Treg suppressive function. We therefore tested the hypothesis that the failure to induce tolerance to topical antigen was due to loss of Treg function in the altered dermal niche. Thus, we applied our tolerance protocol, and 7-day post-challenge CD4^+^CD25^high^CD127^low^ dermal Treg were sorted directly from the skin and tested for their potential to suppress activated CD4^+^CD25^low^CD127^+^ responder T cells isolated from the LNs of untreated mice. The proportion of FoxP3^+^ cells within the CD4^+^CD25^high^ gate was equivalent between BMT and BMT + T groups (59.2 ± 4.63 versus 56.6 ± 10.10 SEM). Figures 6H and 6I show that, while *ex vivo* CD25^high^CD4 T cells from mice that had received BMT, but had not experienced aGVHD, suppressed proliferation of responder T cells, this capacity was lost within Treg isolated from the dermis post-aGVHD. To determine whether we could detect phenotypic differences between Treg from mice that had previously received BMT alone or with T cells, we further analyzed the dermal populations by flow cytometry. Treg from both groups expressed equivalent levels of CTLA-4 (Figure S7A) and ICOS (Figures S7B and S7C), demonstrating that the difference in ICOS expression activated in draining LNs was not maintained in the skin. The integrin CD103 is associated with retention of T cell in the skin and has been shown to be required for the Treg function during murine CHS (Braun et al., 2015). We observed that, while dermal Treg from mice that had previously experienced aGVHD expressed higher levels of the integrin CD103 than Tconv from the same environment, these levels were lower than in Treg from control BMT mice (Figures S7B and S7C). Thus, Treg display a reduced suppressive function within the dermal niche despite clinical recovery from disease.

In summary, we demonstrate the impact of T cell pathology on baseline immunity in the skin and show that parallel breakdown in myeloid and lymphoid compartments is associated with long-term loss of immune homeostasis.

## Discussion

We have used a murine model of hematopoietic stem cell transplantation and aGVHD to determine the impact of immune pathology on the dermal myeloid landscape, and to define the functional long-term consequences of disease on the maintenance of immune homeostasis in the skin. In this model, transferred allo-reactive Mh T cells are recruited to the inflamed skin after BMT, wherein they cause pathology due to killing of recipient cells and release of effector cytokines (Santos et al., 2018). We show that recruitment of monocytes into different skin anatomical compartments leads to the initiation of contrasting transcriptional programs and that, in the aGHVD dermis, this results in the specific expansion of a distinct population of immunomodulatory macrophages in murine and patient skin. Donor macrophages maintain an inflammatory MHCII^high^ profile, fail to restore the resident quiescent MHCII^int^ dermal macrophage compartment, and remain functionally distinct to dermal MHCII^high^ macrophages from mice that did not experience aGHVD. Since immune homeostasis at barrier sites depends on interplay between mononuclear phagocytes and T cells, we tested the functional impact of aGVHD on regulation of T cell immunity in the skin. Exposure of skin to topical antigen 10 weeks post-GVHD resulted in an exaggerated atopic dermatitis and loss of Treg-mediated induction of local tolerance. Cell-cell interaction analysis of human skin suggested that loss of tolerance was due to subversion of Treg function by dermal inflammatory macrophages, and we demonstrated the discrete loss of suppressive activity by Treg isolated from the disturbed dermal niche. Thus, we provide evidence that immune pathology leaves an immunological scar in the dermis in which failure of monocytes to repair the dermal macrophage niche is associated with loss of T cell regulation at the skin immune barrier.

Despite Mh T cell-mediated pathology in both the epidermis and dermis of the skin, we show discrete and contrasting fates for monocyte-derived cells within these juxtaposed sub-compartments; Ly6C^+^ monocytes that enter the epidermis are programmed for cell division, and we have previously shown that they differentiate into quiescent self-renewing LCs that are indistinguishable from the embryonic cells they replace (Ferrer et al., 2019). By contrast, dermal monocytes are specifically primed to become immunomodulatory cells that express classic inflammatory and regulatory cytokines. Such cells have been shown to be key players in the controlled progression of an inflammatory response in the gut (Askenase et al., 2015; Grainger et al., 2013), and typically express factors such as IL-10, and PGE-2 to promote the transition to resolution and tissue repair. Future fate-mapping studies are needed to determine the transition of Ly6C^high^ monocytes entering the skin, but co-upregulation of some genes (cd63, *adam8*, *cxcl16*, *mmp14)* in dermal and epidermal cells compared with circulating blood monocytes points toward activation of Ly6C^+^MHCII^+^ monocytes within the dermis before transition to the epidermis, in which the inflammatory program appears to be downregulated. Notably, these genes were previously identified as core hub genes activated in monocyte-derived cells within the injured lung environment (Misharin et al., 2017), suggesting conservation of the response to tissue pathology between the skin and lungs.

We have recently shown that the dominant expansion of CD11c^+^CD14^+^ macrophages is a defining feature of the immune landscape within skin lesions of patients with aGVHD (Jardine et al., 2020), and here demonstrate homology between these cells and the Ly6C^+^MHCII^+^ dermal differentiated monocytes identified in our murine model. To determine whether expansion of CD11c^+^CD14^+^ macrophages was specific to the aGVHD tissue environment we analyzed single-cell transcriptomics data from human healthy and diseased skin. Parsing myeloid cells revealed all six blood DC populations (Villani et al., 2017) in skin, including Axl^+^Siglec-6^+^ DCs (AS-DC) shown to infiltrate skin during inflammation (Chen et al., 2020). Using a murine/human aGVHD gene signature we identified a cluster of *ITGAX*^*+*^*CD14*^*+*^*S100A9*^*+*^ *PTGS2*^*+*^ macrophages that expanded in psoriatic but not acne skin compared with healthy controls. Thus, accumulation of immunomodulatory monocyte-derived cells appears to be a hallmark of T cell pathologies in the skin.

Donor monocytes differentiated into CCR2^neg^MHCII^high^ dermal macrophages but, compared with BMT controls, these cells failed to rebalance the quiescent CD64^low^MHCII^int^ macrophage compartment that controls homeostasis in healthy skin (Kolter et al., 2019). Current models suggest that differentiation of resident macrophages depends on access to tissue-specific niches (Guilliams and Scott, 2017), and incumbent resident peritoneal macrophages prevent the transition of recruited MHCII^high^ cells to MHCII^low^ macrophages, probably by competing for local niches (Louwe et al., 2021). In our model all resident macrophages are replaced after the combination of irradiation and aGVHD, suggesting that niche accessibility may not be the limiting factor in permitting development of MHCII^int^ cells; rather, chronic inflammation may prevent dermis-specific signals from instructing a resident macrophage fate.

Loss of MHC^low^ interstitial macrophages in the lung is associated with the exacerbation of fibrosis (Chakarov et al., 2019), demonstrating the importance of differentiation to a more quiescent state for the healthy restoration of the tissue environment. It has been proposed that inflammation imprints an activatory gene program in lung monocyte-derived cells, which must be re-set within the lung tissue environment to permit restoration of more quiescent cells (Guilliams and Svedberg, 2021; Kulikauskaite and Wack, 2020). Moreover, following infection in the lung, there may be a window of time in which the presence of local pro-inflammatory monocyte-derived macrophages is beneficial, as recently demonstrated in work from the Wack lab showing that influenza elicits IL-6-producing monocyte-derived alveolar macrophages that are protective against bacterial super-infection (Aegerter et al., 2020). By contrast, dermal MHCII^high^ macrophages expressed increased levels of IL-6 and IL-1β compared with BMT controls beyond the peak of disease, suggesting on-going functional differences in these cells. These data suggest that, in the context of cutaneous aGVHD, inflammation-imprinted macrophages are hard-wired in the dermis, and that the persistence of immune-reactive cells is likely to perpetuate the impact of immune pathology at this site. We note that monocyte-derived alveolar macrophages that differentiated within fibrotic lungs maintained an inflammatory phenotype for up to a year before acquiring a resident macrophage state (Misharin et al., 2017). Thus, longer studies are needed to determine whether the dermal macrophage compartment will ultimately re-set over time.

Crosstalk between mononuclear phagocytes and T cells generates an “axis of tolerance” to maintain immune homeostasis in steady-state gut (Mortha et al., 2014), and to regulate T cell immunity in the skin (Naik et al., 2015). To assess the impact of T cell pathology on immune homeostasis in the skin, we chose a model of tolerance induction to contact sensitizers in which elicitation of local immune responses depends on regulation of T cell function within dermal macrophage clusters (Honda and Kabashima, 2016; Kabashima et al., 2019; Natsuaki et al., 2014). Topical application of haptens to the skin of mice that had recovered from aGVHD failed to elicit Treg-mediated control of effector T cell function, resulting instead in an exaggerated and prolonged atopic dermatitis, and we showed that this was due to direct loss of Treg suppressive function in the dermal environment. We have recently demonstrated the accumulation of Treg in patient aGVHD lesions (Jardine et al., 2020) and here extend these studies to demonstrate discrete functional Treg defects within the dermis that extend beyond the resolution of T cell pathology. A single-cell dataset from cutaneous aGHVD has not currently been published; therefore, in order to understand how alterations in dermal macrophages could impact on local Treg function, we reasoned that investigation of the homologous CD11c^+^CD14^+^ monocyte cell interaction pathways in healthy and psoriatic skin would provide some insight into potentially similar pathways in the context of GVHD. These data emphasized increased signalling via TNFSF members, including loss of ICOS signalling from immunomodulatory CD11c^+^CD14^+^ macrophages to CD4 Treg, and were consistent with the pleiotropic effects of TNF and TNFSF members on Tconv function and Treg dysfunction within the tissue (Wehrens et al., 2013). Additional phenotypic analysis of dermal T cells demonstrated that Treg, but not Tconv, in the skin of mice that had recovered from aGVHD expressed lower levels of CD103. Expression of this integrin has previously been shown to be required for the regulatory function of Treg during murine CHS in addition to promoting retention of T cells in the skin (Braun et al., 2015), suggesting a functional mechanism for the loss of T cell suppression. However, further studies are needed to directly link the production of factors by MHCII^high^CD64^high^ macrophages to local Treg dysfunction in the dermis.

In the disease setting, we have previously shown that epidermal LCs directly regulate allogeneic T cell function and survival, and that the unique transcriptional profile of allogeneic effector T cells in the dermis suggests that local interactions in this environment also regulate a compartment-specific response during on-going aGVHD (Santos et al., 2018). The data presented herein imply that monocyte-derived macrophages that have been primed by the inflammatory aGVHD environment also have an on-going role in shaping the dermal environment beyond the primary disease, and that changes to the macrophage niche impact on third-party endogenous T cells, subsequent to the allogeneic response. Stromal cells within tissues, such as the liver, provide a niche within which macrophages differentiate (Bonnardel et al., 2019), and adaptation of stromal cells to a chronically inflamed environment has been linked to local immune cell dysregulation and autoimmunity under conditions of chronic inflammation (Honan and Chen, 2021). Analysis of secreted proteins in the dermis during aGVHD demonstrated upregulation of factors, such as PDGF-BB, which is a mitogenic factor for fibroblasts. While we did not detect ongoing chronic inflammation in our model, it is important to consider that crosstalk between the stromal and macrophage compartments would contribute to the altered dermal microenvironment and could result in the production of factors that maintain the abundance of MHCII^high^ macrophages.

ICOS^+^ Treg were efficiently primed in the draining LNs of sensitized mice and trafficked to the skin at the challenge site. Accumulation of hyper-activated Treg is a characteristic of sites of tissue auto-inflammation and -immunity (Pesenacker et al., 2015). Moreover, recent studies have highlighted the adaptability of Treg to the local tissue environment (Whibley et al., 2019); and multiple inflammatory cytokines trigger the pathogenic conversion of CD4 Treg to inflammatory Tconv-like cells, such as in psoriatic skin (Kannan et al., 2019), genetic reprogramming of FoxP3^+^ Treg (Yang et al., 2008), and Treg dysfunction (Pesenacker et al., 2015). Together, our data suggest a model in which direct and indirect interactions between monocyte-derived macrophages and dermal T cells results in both skewing of the ratio of Tconv:Treg cells, and direct loss of suppressive function by Treg, ensuring that the regulatory machinery is overwhelmed within the altered dermal niche. A recent single-cell sequencing study has highlighted the heterogeneity within cutaneous Treg during CHS, and the plasticity of some Treg markers within the tissue environment. Future work using fate-mapping reporter mouse models is needed to determine the extent to which thymic and peripheral Treg are recruited and/or accumulate in the dermis after aGVHD, and how these populations are impacted by the altered dermal environment.

In summary our data show that macrophage homeostasis within the dermis is highly sensitive to immune pathology, and that failure to restore balance is associated with long-term loss of T cell regulation. Our current study advances a growing understanding of the importance of skin macrophages in promoting pathology by suggesting that inflammation-induced programming of monocyte-derived cells leads to sustained immune disequilibrium in the skin. Collectively our findings shed light on the need to consider the long-term impact of immunotherapies on patients beyond recovery from primary disease.

## STAR★Methods

### Key resources table

**Table:** 

REAGENT or RESOURCE	SOURCE	IDENTIFIER
**Antibodies**		

Anti-CD24 FITC clone M1/69	BioLegend	Cat # 101805; RRID: AB_312838
Anti-CD11b e450	eBioscience	Cat# 48-0112-82; RRID: AB_1582236
Anti-Ly6C PeCy7 clone AL-21	BD Biosciences	Cat # 560593; RRID: AB_1727557
Anti-CD64 BV711clone x54-5.7.1	BioLegend	Cat # 139311; RRID: AB_2563846
Anti-CD64 PE clone x54-5.7.1	BioLegend	Cat # 139303; RRID: AB_10613467
Anti-CCR2-APC clone 475301	R&D Systems	Cat# FAB5538P
Anti-MHCII I-A/I-E v500 clone M5/114	BD Biosciences	Cat# 562366; RRID: AB_11153488
Anti-CD3 APCCy7 clone 145-2C11	BD Biosciences	Cat# 561042; RRID: AB_396759
Anti-CD19 APCCy7 clone1D3	BD Biosciences	Cat # 557655; RRID: AB_396770
Anti-NK1.1 APCCy7 clone PK136	BD Biosciences	Cat # 560618; RRID: AB_1727569
Anti-Ly6G APCCy7 clone 1A8	BD Biosciences	Cat # 560600; RRID: AB_1727561
Anti-CD45.1 BV650 clone A20	BD Biosciences	Cat# 563754; RRID: AB_2738405
Anti-CD45.2 PerCP-Cy5.5 clone 104	eBioscience	Cat# 45-0454-82; RRID: AB_2534956
Anti-CD45.2 APCCy7 clone 104	BD Biosciences	Cat # 560694; RRID: AB_1727492
Anti-CD3 APC clone 145-2C11	BD Biosciences	Cat # 553066; RRID: AB_398529
Anti-CD4 PE clone GK1.5	eBioscience	Cat # 557308; RRID: AB_396634
Anti-CD8 v450 clone 53-6.7	BD Biosciences	Cat# 560471; RRID: AB_1645281
Anti-V beta 8.3 FITC clone	BD Biosciences	Cat # 553663; RRID: AB_394979
Anti-CD5 APC clone 1B3.3	eBioscience	Cat# 17-0051-82; RRID: AB_469331
Anti-CD8 BUV395 clone53-6.7	BD Biosciences	Cat# 565968; RRID: AB_2732919
Anti-CD25 BV786 clone PC61	BD Biosciences	Cat# 564023; RRID: AB_2738548
Anti-CD25 APC clone PC61	BD Biosciences	Cat # 561048; RRID: AB_398623
Anti-gamma delta TCR PE clone eBioGL3	eBioscience	Cat # 12-5711-82; RRID: AB_465934
Anti-NK1.1 PE clone PK136	BioLegend	Cat # 108707; RRID: AB_313394
Anti-CD11b PE clone M1/70	eBioscience	Cat # 12-5711-82; RRID: AB_465934
Anti-CD4 BUV737 clone GK1.5	BD Horizon	Cat # 612761
Anti-CD3 PE Cy7 clone 145-2C11	BD Biosciences	Cat # 561100; RRID: AB_394460
Anti-CD4 FITC clone GK1.5	eBioscience	Cat # 553729; RRID: AB_395013
Anti-ICOS BV421 clone C398.4A	BD Biosciences	Cat # 565886; RRID: AB_2869726
Anti-ICOS BUV395 clone C398.4A	BD Bioscience	Cat # 565885; RRID: AB_2744481
Anti-CD69 PeCy7 clone H1.2F3	eBioscience	Cat # A16360; RRID: AB_2534952
Anti-CD103 APC R700 clone M290	BD Bioscience	Cat # 565529; RRID: AB_2739282
Anti-FoxP3 APC clone FJK-165	eBioscience	Cat # 14-5773-82; RRID: AB_467576
Anti-CTLA4 PE-Dazzle clone UC10-4B9	Biolegend	Cat# 106317; RRID: AB_2564495
Anti-IL-6 PE clone MP5-20F3	eBioscience	Cat # 12-7061-41; RRID: AB_1633409
Anti-TNFa PE clone MP6-XP22	Biolegend	Cat # 506305; RRID: AB_315426

**Chemicals, peptides, and recombinant proteins**		

Dispase 2	Roche (Thermofisher)	Cat # 04942078001
Collegenase IV from Clostridium histolyticum	Worthington	Cat # NC9919937
Fixable viability dye eFluor780	eBioscience	Cat # 65-0865-18
Fixable viability dye e450	eBioscience	Cat # 65-0863-14
CountBright plus absolute counting beads	Thermofisher	Cat # C36995; CAS 70-34-8
CellTrace Violet	Thermofisher	Cat # C34557
2,4-dinitrofluorobenzene (DNFB)	Fisher Scientific	Cat # 10724041
2,4-dinitrothiocyanobenzene (DNTB)	Fisher Scientific Alfa Aesar	Cat # 11421167;CAS 594-56-5
MAXIMA SYBER GREEN/ROX qPCR 2X	VWR	Cat # K0221

**Critical commercial assays**		

Proteome Profiler Mouse XL Cytokine Array	R&D systems	Cat # ARY028
BD Fixation/Permeabilization Kit	BD Biosciences	Cat # 554714
High capacity DNA reverse transcription kit	Qiagen / Life Technologies	Cat # 4368814

**Deposited data**		

Gene Expression Omnibue (GEO): GSE150672	Hughes et al.	https://doi.org/10.1016/j.immuni.2020.09.015
Gene Expression Omnibue (GEO): GSE130257	Ferrer et al.	https://doi.org/10.1126/sciimmunol.aax8704
Gene Expression Omnibue (GEO): GSE94820	Villani et al.	https://doi.org/10.1126/science.aah4573.

**Experimental models: Organisms/strains**		

C5B7BL/6NCrl	Charles River or bred in house	N/A
CD45.1 mice (B6.SJL-*Ptprc*^*a*^*Pepc*^*b*^/BoyJ)	Bred in house	RRID:IMSR_JAX:002014
Matahari mice (Tg(TcraMataHari,TcrbMatahari)#Lantz)	Bred in house	MGI:5829381

**Oligonucleotides**		

Murine forward TNFa primer for qRT-PCR: CAGGCGGTGCCTATGTCTC	Thermofisher	Primerbank ID: 133892368c1
Murine reverse TNFa primer for qRT-PCR: CGATCACCCCGAAGTTCAGTAG	Thermofisher	Primerbank ID: 133892368c1
Murine forward IL1b primer for qRT-PCR: GAAATGCCACCTTTTGACAGTG	Thermofisher	Primerbank ID: 118130747c1
Murine reverse IL1b primer for qRT-PCR: TGGATGCTCTCATCAGGACAG	Thermofisher	Primerbank ID: 118130747c1
Murine forward beta actin primer for qRT-PCR: GGCTGTATTCCCCTCCATCG	Thermofisher	Primerbank ID: 6671509a1
Murine reverse beta actin primer for qRT-PCR: CCAGTTGGTAACAATGCCATGT	Thermofisher	Primerbank ID: 6671509a1

**Software and algorithms**		

FlowJo v9 and 10	Tree Star, LLC USA	https://www.flowjo.com/
Prism v6,8 and 9	GraphPad	https://www.graphpad.com/
CellPhoneDB	N/A	https://www.cellphonedb.org/
QuPath (Quantitative Pathology & Bioimage Analysis)	N/A	https://qupath.github.io/

### Resource availability

#### Lead contact

Further information and requests for resources and reagents should be directed to and will be fulfilled by the lead contact, Clare Bennett (c.bennett@ucl.ac.uk).

#### Materials availability

This study did not generate new unique reagents.

### Experimental model and subject details

#### Animal models

CD45.2 C57BL/6 (B6) mice and CD45.1 congenic controls were purchased from Charles River and/or bred in house in the UCL Comparative Biology Unit. C57BL/6 TCR-transgenic anti-HY MataHari mice (Valujskikh et al., 2002) were provided by Jian Chai (Imperial College London, London, UK). Male mice (10+ weeks and/or weighing at least 20g) were used as transplant recipients for all experiments and were randomly assigned to BMT and BMT + T treatment groups. Untreated mice and tissue donors were female mice (6–10 weeks old). Mice were housed in specific pathogen free conditions. All procedures were conducted in accordance with the UK Home Office Animals (Scientific Procedure) Act of 1986, and were approved by the Ethics and Welfare Committee of the Comparative Biology Unit, Hampstead Campus, UCL, London, UK.

### Method details

#### Bone marrow transplants (BMT)

Recipient male CD45.2 B6 mice were lethally irradiated (11 Gy total body irradiation, split into 2 fractions over a period of 48 h) and reconstituted 4 h after the second dose with 5 × 10^6^ female CD45.1 C57BL/6 BM cells and 2 × 10^6^ CD4 T cells, with 1 × 10^6^ CD8 Thy1.1^+^ Matahari T cells, administered by intravenous injection through the tail vein. CD4 and CD8 T cells were isolated by magnetic selection of CD4 or CD8 splenocytes using the Miltneyi MACS system (QuadroMACS Separator, LS columns, CD4 [L3T4] MicroBeads, CD8a [Ly-2] MicroBeads; Miltenyi Biotec) for injection of splenic T cells. In some experiments syngeneic controls were use in which female recipients received female BM with Matahari T cells.

#### Induction of tolerance/contact hypersensitivity (CHS)

Mice were sensitised epicutaneously on day 0 by application of 25 μL 0.5% 2,4-dinitrofluorobenzene (DNFB; Fischer Scientific) in acetone/olive oil (4:1 v/v) onto 2cm^2^ shaved abdominal skin. Mice were challenged on day 5 with a topical application of 10μL of a non-irritant concentration of 0.2% DNFB in acetone/olive oil to the right ear. The left ear was treated with acetone/olive oil alone. Mice that were ear challenged without previous sensitisation served as non-specific inflammation controls. To induce cutaneous tolerance, mice received 100μL topical 1% 2,4-dinitrothiocyanobenzene (DNTB; Fisher Scientific Alfa Aesar) in acetone/olive oil onto 2cm^2^ shaved flank skin 7 days prior to sensitisation. Ear thickness was measured blinded using a digital caliper and calculated as (T-T0 of the right ear) - (T-T0 of the left ear), where T0 and T represent the values of ear thickness before and after the challenge, respectively.

#### Proteome profiling

Ears were excised, split into dorsal and ventral sides and incubated on 2.5mg/mL dispase II (Roche) at 37°C for 45 min to permit separation of epidermis and dermis. Dermal sheets were pooled from 2 mice per group were then floated separately on 1mL RPMI supplemented with 2% heat-inactivated fetal calf serum at 37°C for 16 h. Supernatant was assayed for the presence of 111 cytokines using the Proteome Profiler Mouse XL Cytokine Array (R&D Systems), according to manufacturer’s instructions. Data shown are proteins unregulated >2 fold in epidermis and >1.2 fold in dermis in mice receiving BMT with T cells compared to BMT only controls.

#### Generation of tissue single cell suspensions

##### Ear skin

Dermis was separated as described for the proteome profiling, cut into small pieces and digested with 250 U/mL collagenase IV (Worthington) and 800U/mL DNaseI (Roche) at 37°C for 1 h. Dermal single cell suspensions were then generated using the GentleMACS tissue dissociator (Miltneyi Biotech).

##### Lymph nodes (LN)

Draining LN were mechanically disrupted in MACS buffer (1% heat-inactivated fetal calf serum and 1 mM EDTA (Sigma-Aldrich) in PBS) using a syringe plunger. The cells were filtered through nylon mesh.

#### Flow cytometry

All antibodies used are summarised in the Key resources table. Cells were distributed in 96 well conical bottom plates and incubated in 2.4G2 hybridoma supernatant (containing αCD16/32) for at least 10 min at 4°C to block Fc receptors. For cell surface labeling, cells were incubated with fluorochrome-conjugated antibodies diluted in 100μL FACS buffer (PBS/1mM EDTA/1% heat-inactivated fetal calf serum) at 4°C for at least 20 min in the dark. To exclude lineage^+^ cells, we used a cocktail of antibodies against CD3, CD19, NK1.1 and Ly6G all conjugated to APC-Cy7. Antibodies to define dermal T cells are described in Figure S5. Nuclear FoxP3 and CTLA-4 were detected using the eBiosciences FoxP3/Transcription factor staining buffer kit.

For detection of *ex vivo* cytokines, cells were treated with brefeldin A (Sigma-Aldrich) for 2 h at 37°C before washing in FACS buffer and immunolabelling with surface. Samples were washed twice with FACS buffer and re-suspended in 300 μL of FACS buffer for immediate analysis by flow cytometry Intracellular staining was performed after cell surface immunolabelling. To label intracellular cytokine, cells were fixed in 100μL fix solution (BD Cytofix/Cytoperm solution, BD Biosciences) for 15 min at 4°C, washed twice with permeabilisation buffer (BD Perm/Wash, BD Biosciences) and incubated with 100μL of the antibody diluted in permeabilisation solution at 4°C for 30 min in the dark.

Live cells were identified by exclusion of propidium iodide (unfixed cells), or a fixable viability dye (eBioscience). Absolute number of dermal T cells was determined by flow cytometry using CountBright™ Absolute Counting Beads (Thermofisher Scientific), as per the manufacturer’s instructions.

Multicolor flow cytometry data were acquired with BD LSRFortessa and BD LSR II cell analyzers equipped with BD FACSDiva software. Flow cytometry data were analyzed with FlowJo X v9 and 10 (LLC USA), and live cells were pre-gated on singlets (FSC-A versus FSC-H), and a morphological FSC/SSC gate.

#### Histology

##### Ear skin

Portions of ear skin were excised from mice that had received BMT with T cells 10 weeks earlier, or age-matched untreated controls, and snap frozen in OCT. Cryosections were subsequently stained with haematoxylin and eosin or Masson’s Trichrome. Slides were scanned using the Nanozoomer (Hamamatsu) and epidermal thickness quantified blind using the morphology module in QuPath (Quantitative Pathology & Bioimage Analysis).

##### Salivary glands

Sub-mandibular salivary glands were isolated, fixed in 4% para-formaldehyde and frozen in OCT. Cryosections were stained with haematoxylin and eosin and assessed blind according to (Groom et al., 2002): 1 = 1–5 foci of mononuclear cells seen (more than 20 cells per focus); 2 = <5 foci of mononuclear cells, without significant parenchymal destruction; 3 = multiple confluent foci and moderate degeneration of parenchymal tissue; 4 = extensive infiltration and extensive parenchymal destruction.

#### Suppression assay

The suppression assay was based on a published protocol (Collison and Vignali, 2011). Mice were exposed to the tolerance, sensitisation and challenge protocol. 7 days post-challenge both ears were harvested and five mice per group were pooled. CD4^+^CD25^+^ cells were sorted by flow cytometry from single cell suspensions. These were cultured at various ratios with CellTrace™ violet (CTV, Thermofisher Scientific)-labelled sorted CD4^+^CD25^neg^CD127^+^ responder T cells from the LN of untreated mice in the presence of anti-CD3 and splenic CD19^+^ B cells for 72 h. Proliferation of CTV-labelled responders was tracked by flow cytometry.

#### Quantitative RT-PCR

RNA from snap-frozen sorted MHCII^high^CD64^high^ dermal macrophages was isolated using the Qiagen micro RNeasy kit. RNA was reverse transcribed to cDNA with a high capacity RNA reverse transcription kit (Life technologies). qRT-PCR was performed on a QuantStudio 5 Real-Time PCR System (Thermofisher Scientific) using SYBR Green (VWR) and the primers listed in the key resources table.

#### RNA expression analyses

##### Murine bulk RNAseq data

Gene expression comparisons from sorted CD11b^+^Ly6C^high^CD115^+^ blood monocytes, CD24^neg^CCR2^+^Ly6C^+^MHCII^+^ dermal activated monocytes and CD11b^+^MHCII^+^CD24^low^ Langerin^neg^EpCAM^+^ epidermal monocyte-derived cells were performed using our published data set that is deposited in the NCBI Gene Expression Omnibus database (GSE130257) (Ferrer et al., 2019). DESeq2 normalised TPM expression data was used for the plots in Figure 1 (Love et al., 2014) with the additional use of variance stabilising transform (vst) of the data for Figure 1F. Gene Set Enrichment Analysis (GSEA) was performed using the MsigDB pathways database from the Broad Institute (Subramanian et al., 2005). DESeq2 differential expression analysis was used to compare CD24^neg^CCR2^+^Ly6C^+^MHCII^+^ dermal activated monocytes and CD11b^+^MHCII^+^CD24^low^ Langerin^neg^EpCAM^+^ epidermal monocyte-derived cells (B&H p < 0.05, logFC>1). Gene expression graphs are shown as relative FPKM counts were normalised to the maximum.

##### Comparison to human aGVHD monocytes

Nanostring gene expression data of human CD11c^+^CD14^+^ cells and blood CD14^+^ monocytes isolated from GVHD patients was generated as previously described (Jardine et al., 2020). Comparative analysis with murine CD11b^+^Ly6C^high^CD115^+^ blood monocytes and CD24^neg^CCR2^+^Ly6C^+^MHCII^+^ dermal activated GVHD monocytes was performed within the R environment. In each respective dataset, DEG analysis (DESeq2, B&H p < 0.05, logFC>1) comparing blood to dermal monocytes was performed. DEGs mutually differentially regulated in both human and murine analyses were identified, with heatmap plotting used to display overlap in blood monocyte or dermal monocytes upregulated genes in each respective dataset. Hypergeometric testing to assess overlap was performed using the GeneOverlap R package.

##### Single cell RNA sequencing analysis

Publicly available single cell gene expression data from (Hughes et al., 2020) was acquired from GEO (GSE150672). The python-based Scanpy analysis pipeline (version 1.5.0) was used (Wolf et al., 2018). Data from individual samples was first extracted. Low quality cells, with a high fraction of counts from mitochondrial genes (20% or more) indicating stressed or dying cells were removed. In addition, genes with expression detected in <10 cells were excluded. Datasets were normalised using scran (Lun et al., 2016), using rpy2 within python. Highly variable genes (top 2000) were selected using distribution criteria: min_mean = 0, max_mean = 4, min_disp = 0.1. A single-cell neighborhood graph, with integrated data from individual samples, was computed using BBKNN (Polanski et al., 2020). Uniform Manifold Approximation and Projection (UMAP) was performed for dimensionality reduction, with the Leiden algorithm (Traag et al., 2019) used to identify clusters within cell populations (Leiden r = 0.5). Differentially expressed genes (DEGs) between cell clusters were identified using t test within scanpy (FDR corrected p value<0.01, logFC>1). Marker genes for cell clusters were identified using a t test. SingleR automatic cell type annotation was performed using BlueprintEncodeData, HumanPrimaryCellAtlasData and DatabaseImmuneCellExpressionData references (Aran et al., 2019). Investigation into the enrichment of DC subpopulations was performed using blood DC signatures obtained from Villani et al. (GSE94820) (Villani et al., 2017).

##### Receptor-ligand interaction analysis

Scran normalized single cell expression values of CD14^+^CD11c^+^ Cluster 1 Monocytes and CD4^+^ Tregs from normal and psoriatic skin were exported from Scanpy. Cell-cell network interaction inference analysis, based on the expression of compatible ligands and receptors between compared cell types, was performed using CellPhoneDB (Efremova et al., 2020). Visualisation and comparative analysis of psoriasis versus normal CD14^+^CD11c^+^ Cluster 1 monocytes and CD4^+^ Treg cellular interactions was performed using CrossTalkeR (Nagai et al., 2021).

### Quantification and statistical analysis

All data apart from RNAseq data were analysed using GraphPad Prism for Mac OsX (GraphPad Software, USA). All line graphs and bar charts are expressed as means ± SD. Protein expression data for flow cytometry is shown as median or geometric mean fluorescent intensity (as specified in figure legends) with the range. Significance was determined using one-way analysis of variance (ANOVA) to measure a single variable in three groups or two-way ANOVA for experiments with more than one variable, with post-tests as specified in individual figures. For comparisons between 2 unpaired groups an unpaired t test, Mann Whitney was used according to a normality test. A paired Wilcoxon test was used to compare challenged and unchallenged ear thickness in Figure 6. Significance was defined as ^∗^p < 0.05, ^∗∗^p < 0.01, ^∗∗∗^p < 0.001. Statistical details of the data can be found in each figure legend.

Analysis of bulk and single cell RNAseq data was performed in the R and Python environments using tests described in the Method details.

## Data Availability

• All single-cell RNA-seq data and Nanostring data have been reported previously. Accession numbers are listed in the key resources table. Microscopy data reported in this paper will be shared by the lead contact upon request. • This paper does not report original code. • Any additional information required to reanalyse the data reported in this paper is available from the lead contact upon request.

## Supplemental information

Document S1. Figures S1–S7

Table S1. Differential gene expression for murine dermal and epidermal monocyte-derived cells, related to Figure 1

Table S2. Rank differential gene expression data for blood and dermal monocytes from mouse and human, related to Figure 2

Table S3. List of genes used to identify human DC populations in the skin, related to Figure 2

Table S4. Receptor-ligand interaction table for CD11c+C14+ C1 monocytes and Treg, related to Figure 6

Document S2. Article plus supplemental information
