# Supplementary material for: Loss of T cell tolerance in the skin following immunopathology is linked to failed restoration of the dermal niche by recruited macrophages
Source: Cell Rep. 2022 May 17;39(7):110819. doi: 10.1016/j.celrep.2022.110819 (PMC9620741; doi:10.1016/j.celrep.2022.110819)
Supplement: Document S1. Figures S1–S7 [file mmc1.pdf]

Cell Reports, Volume *39*

Supplemental information

Loss of T cell tolerance in the skin following immunopathology is linked to failed restoration of the dermal niche by recruited macrophages

Heather C. West, James Davies, Stephen Henderson, Oluyori K. Adegun, Sophie Ward, Ivana R. Ferrer, Chanidapa A. Tye, Andres F. Vallejo, Laura Jardine, Matthew Collin, Marta E. Polak, and Clare L. Bennett

Supplemental figures

Figure S1. Characterisation of the aGVHD model.

Figure S2. Comparison of epidermal and dermal monocyte fates.

Figure S3. Analysis of single cell dataset from human skin.

Figure S4. Defining donor dermal monocytes.

Figure S5.Gating strategy for dermal T cell populations.

Figure S6. Receptor-Ligand interactions between $CD11c^{+}C14^{+}$ C1 macrophages and Treg.

Figure S7. Phenotypic characterisation of dermal Treg.

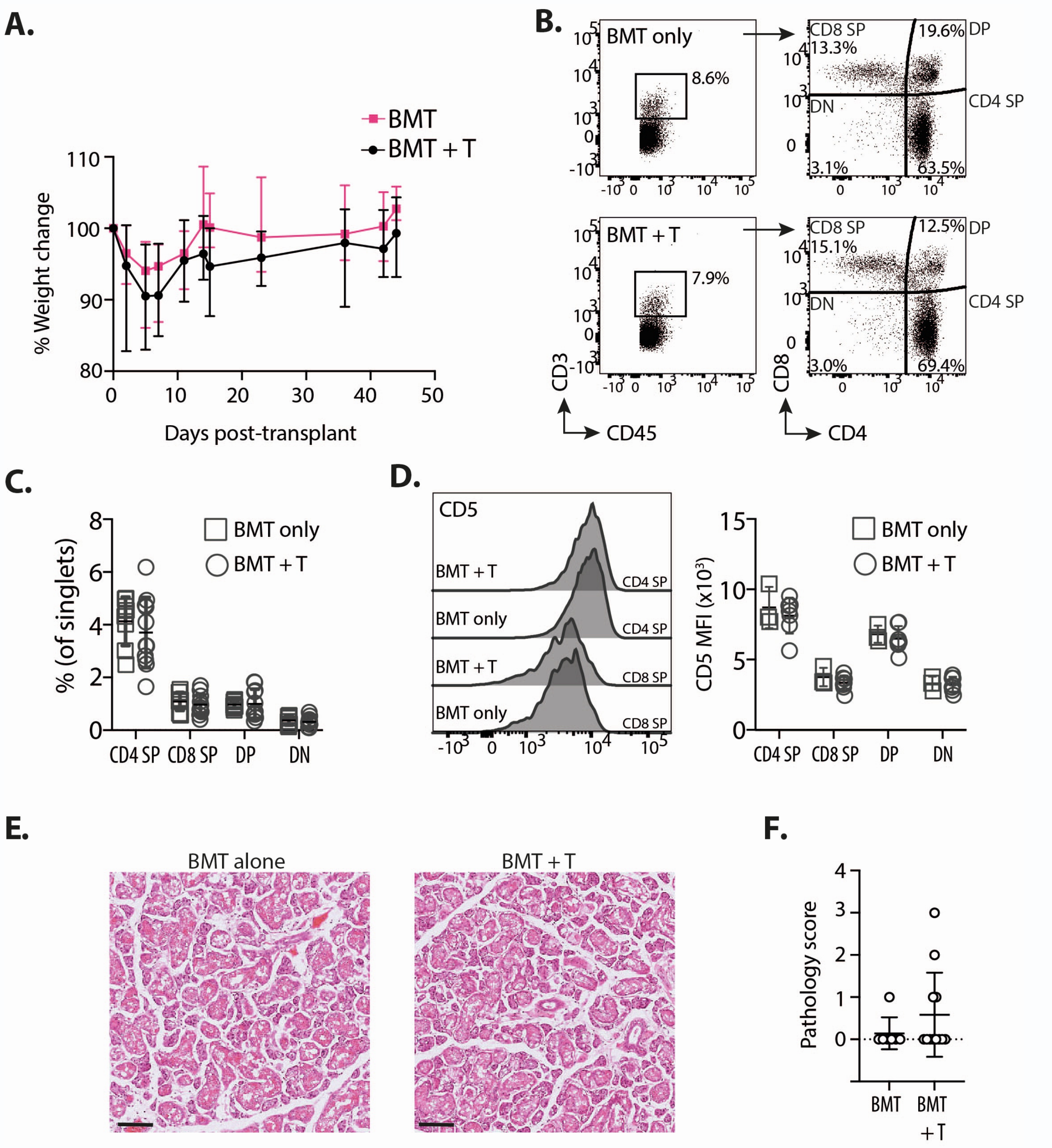

Figure S1. Characterisation of the aGVHD model, related to Figures 1 and 3. A. Graph shows the change in weight over time of male recipients receiving either female syngeneic bone marrow alone (BMT) or bone marrow with T cells (BMT + T). Data points show the mean and range for 14 mice per group pooled from 2 independent experiments. **B.** Representative dot plots plots showing gating of thymic $CD3^{+}CD4^{+}$ and $CD8^{+}$ T cell populations from mice that had received BMT alone or with Mh T cells. **C.** Summary graph showing the frequency of T cell populations from the thymi of transplanted mice 6 to 7 weeks post-transplant; SP = single positive, DP - double positive, DN = double negative. Symbols show individual mice, with lines marking the mean ± SD (n = 8-11 from 2 independent experiments). **D.** *Left* - Representative histograms showing expression of CD5 on gated thymic T cells. *Right* - Summary graph showing the CD5 mean fluorescent intensity (MFI) on thymic T cells 10 weeks post-transplant; SP = single positive, DP - double positive, DN = double negative Symbols show individual mice, with lines marking the mean ± SD; n= 3 BMT and 7 BMT + T from 1 experiment. **E.** *Left* - Representative H&E-stained microscopy images of sub-mandibular salivary glands 6-7 weeks post-transplant with (BMT + T) or without (BMT) T cells. Scale bars = 500µm. *Right* - summary graph showing scoring (with mean ± SD) of images, BMT n=7, BMT + T n=12 from 2 independent experiments; Mann-Whitney P=0.46.

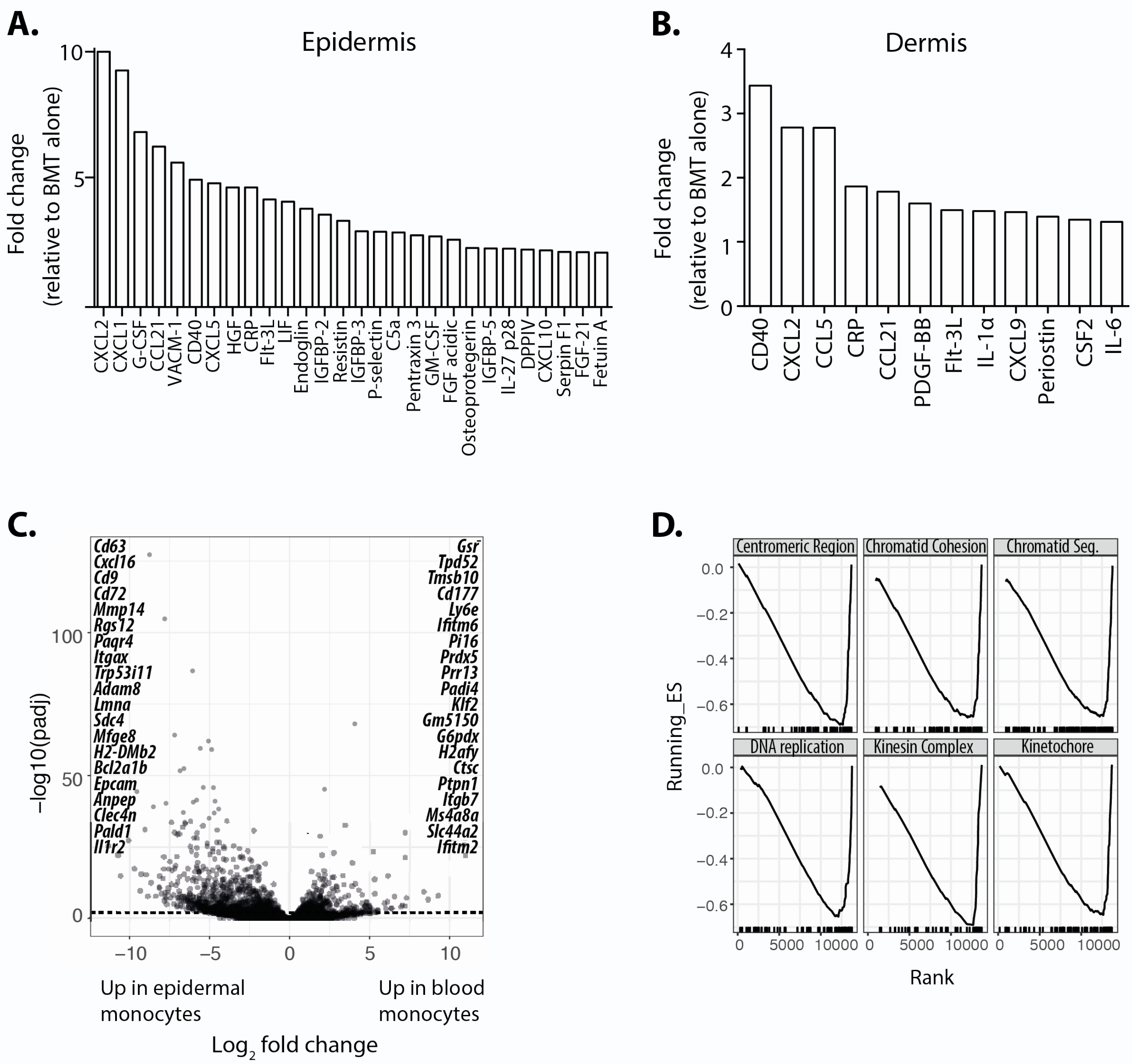

Figure S2. Comparison of epidermal and dermal monocyte fates, related to Figure 1. A and B, . A proteomic screen was performed for secreted epidermal or dermal proteins 3 weeks post-BMT, with or without T cells. The bar graphs show proteins up-regulated at least 2 fold in the epidermis **(A)** and 1.2 fold in the dermis. **(B)** in the setting of BMT with T cells relative to BMT only controls. The tissue was pooled from 2 mice per group. **C.** Volcano plot highlighting the top genes up-regulated in epidermal versus blood monocytic cells. Genes with $Log_2(FC)>\pm2$ and FDR adjusted p-value less than 0.01 were considered significant. **D.** Graphical output from gene set enrichment analysis showing enriched gene ontogeny sets in epidermal compared to blood monocytes.

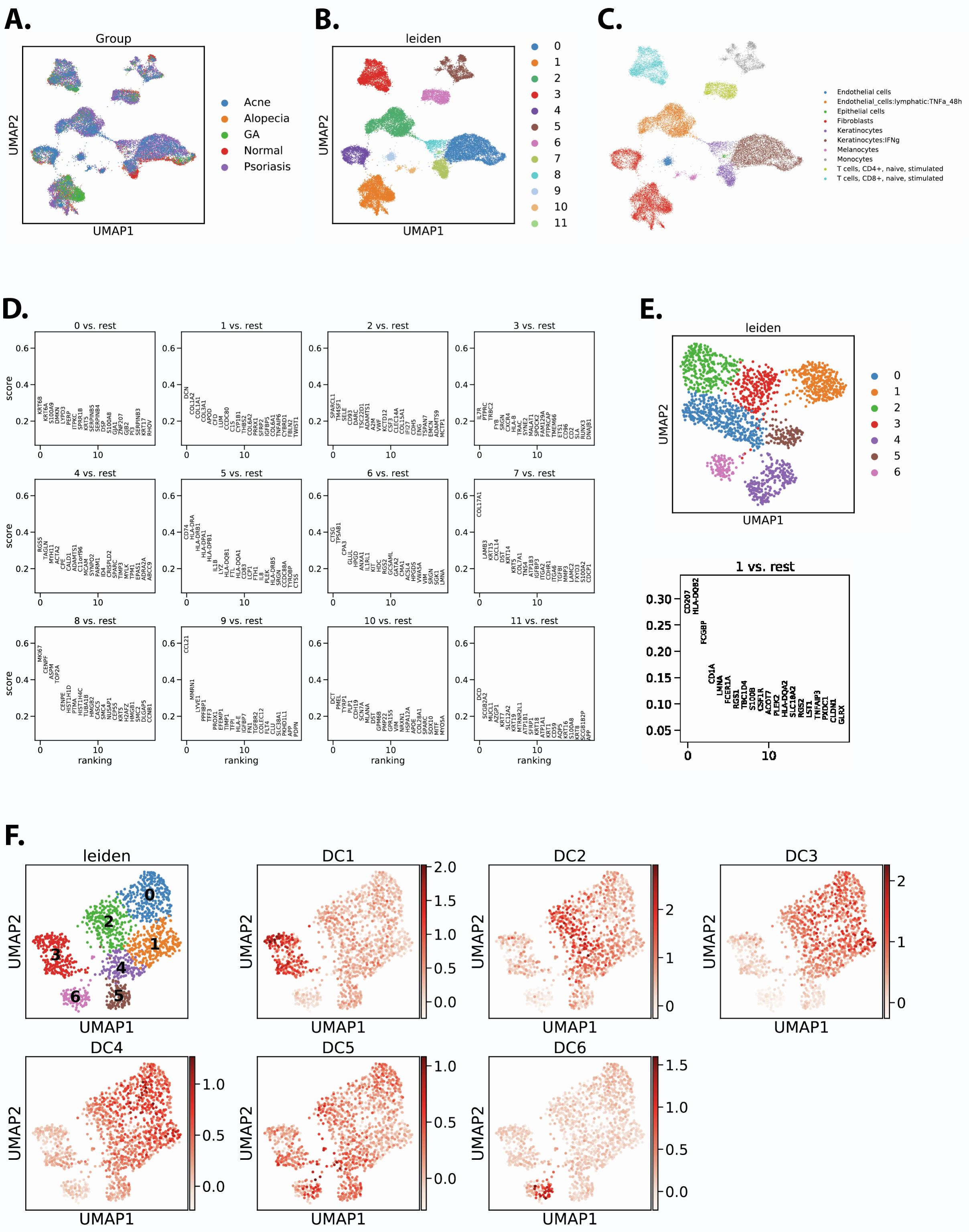

Figure S3. Analysis of single cell dataset from human skin, related to Figure 2. A-F Analysis of human skin single cell data from Hughes *et al*. (GSE150672), comprising whole skin from healthy donors and psoriasis, acne, granulomatous annuloma (GA) and alopecia patients. **A.** UMAP displaying the whole skin populations coloured by disease state. **B.** UMAP displaying the 11 populations identified through unsupervised leiden clustering (r=0.5). **C.** SingleR database annotation [databases: BlueprintEncodeData, HumanPrimaryCellAtlasData and Database Immune Cell Expression Data] identified 10 distinct cell types amongst the singe cell data, including monocyte-related/myeloid cells (cluster 5). **D.** Top 20 marker genes (log regression within Scanpy) for the 11 populations identified through leiden. **E.** UMAP of the subsetted monocyte/cluster 5 population from B. Marker plot (log regression) identified LCs as cluster 1 cells due to elevated expression of *CD207* and *CD1A.* **F.** UMAP plots displaying enrichment of DC1, DC2, DC3, DC4, DC5 and DC6 markers amongst the monocyte/cluster 5 group. Marker genes are those characterised in Villani et al. 2017 (see also Table 3).

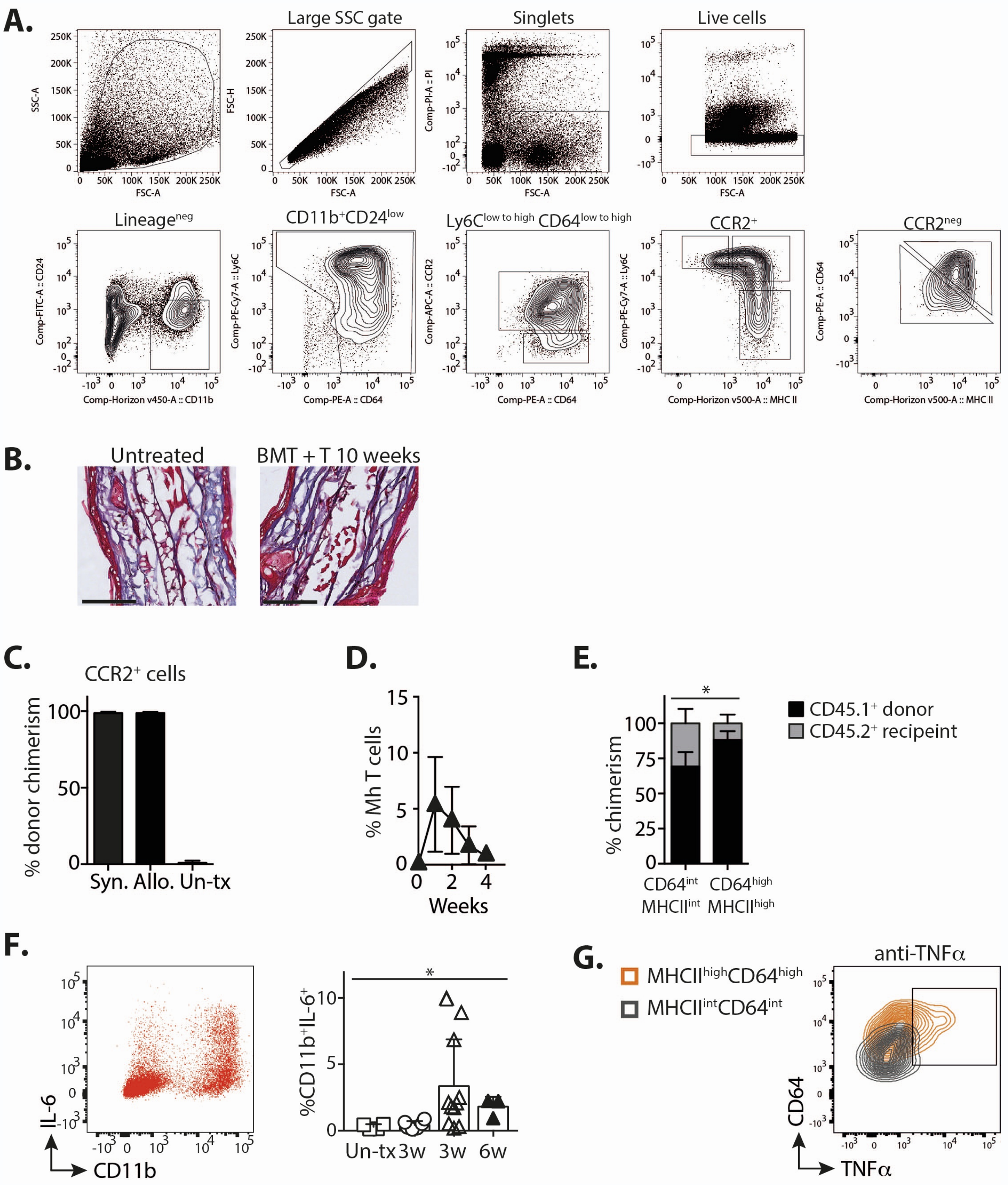

Figure S4. Defining donor dermal monocytes, related to Figure 3. A. Representative flow plots show the gating strategy to identify dermal myeloid cells. **B.** Representative images of ear sections from age-matched untreated mice or recipients of BMT with T cells (10 weeks post-transplant) stained with Masson's Trichrome; scale bars = 100µm. **C.** Summary bar graph shows the donor chimerism (% CD45.1$^+$ cells ± SD) in CCR2$^+$ monocytes 10 weeks post-transplant. Data are pooled from 2 independent experiments, untreated (un-tx) n=3, syngeneic transplant (syn.) n=7, allogeneic transplant (allo.) n-9. **D.** Graph showing the frequency ±SD of dermal Vβ8.3$^+$ Mh T cells at different times post-transplant; n= 7. **E.** Stacked bar graph shows the frequency ±SD of CD45.1 and CD45.2 cells within CCR2^{neg} gated macrophage populations in the dermis of mice receiving BMT with Mh T cells 10 weeks earlier. % CD45.1 cells in CD64^{int}MHCII^{int} versus CD64^{high}MHCII^{high} populations *P*=.029, Mann-Whitney. **F.** *Left* - The representative dot plot showing detection of intracellular IL-6 directly *ex vivo* in dermal myeloid cells. *Right* - Summary graph showing the frequency ±SD of IL-6$^+$CD11b$^+$ cells of lineage negative cells. Symbols are individual mice: untreated n=4; BMT 3 weeks (3w) n=6, BMT + T 3 weeks (3w) n=11 and 6 weeks (6w) n=3. Data are pooled from 2 independent experiments, and analysed by 1-way ANOVA. **G.** Representative contour plots showing TNFa expression by pre-gated CCR2^{neg}MHCII^{high}CD64^{high} and CCR2^{neg}MHCII^{int}CD64^{int} cells. *P<0.05, **P<0.01, ***P<0.001.

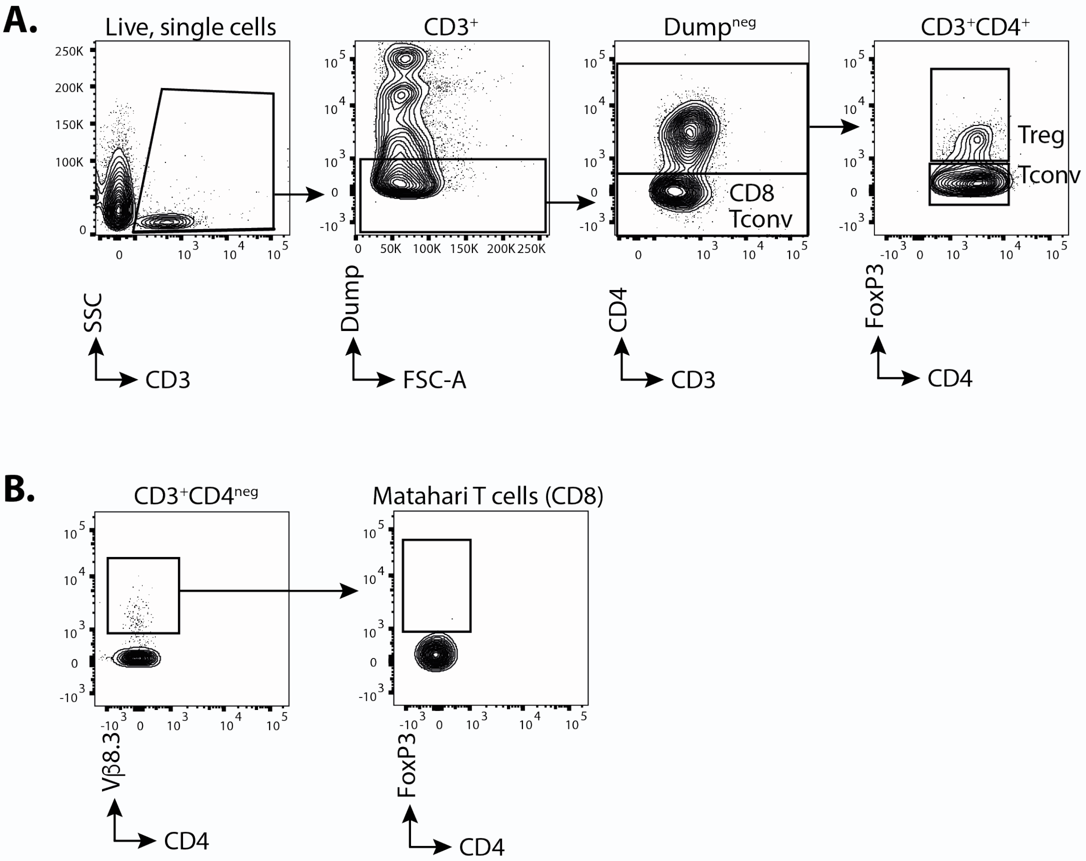

Figure S5. Gating strategy to identify dermal T cells, related to Figure 6. A. Representative contour plots show the gating strategy used to identify $CD3^+CD4^+FoxP3^{neg}$ Tconv and $CD3^+CD4^+FoxP3^+$Treg and $CD3^+CD4^{neg}$ CD8 Tconv. The dump channel was used to exclude contaminating γδ T cells (anti-γδ TCR), myeloid cells (anti-CD64 and anti-CD11b) and NK cells (anti-NK1.1). **B.** Contour plots show the gating of $CD3^+CD4^{neg}$vβ8.3 Matahari T cells. These cells were used as a negative control to set the FoxP3 gate.

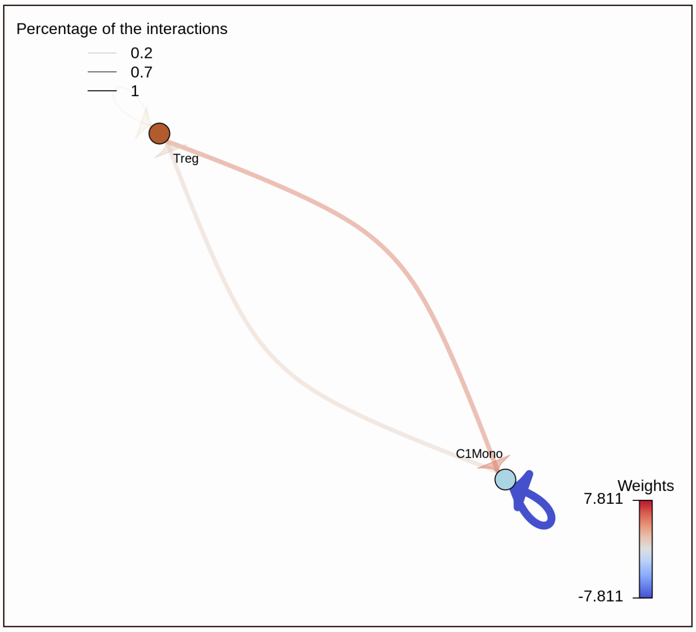

Figure S6. Receptor-Ligand interactions between CD11c⁺C14⁺ C1 macrophages and Treg, related to Figure 6. Cell-cell interaction (CCI) plot displaying the difference in the percentage of receptor-ligand interactions when comparing psoriasis versus normal CD11c⁺CD14⁺ C1 macrophages and Tregs. Red arrows represent edges in which the overall number of receptor-ligand interaction's is increased in psoriasis versus normal, whilst blue arrows represent edges upregulated in normal versus psoriasis. Line thickness represents the percentage of all receptor-ligand interactions taken up by each individual edge.

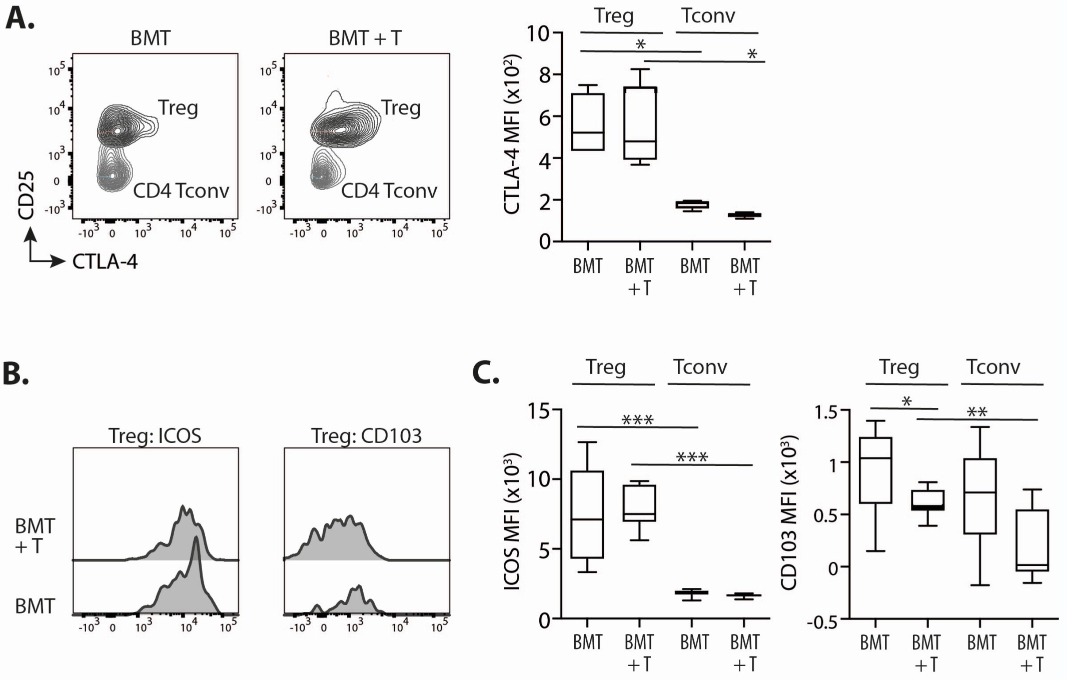

Figure S7. Phenotypic characterisation of dermal Treg, related to Figure 6. Mice received bone marrow transplants without (BMT) or with T cells (BMT+T). 8 weeks later they were treated with topical haptens according to the tolerising protocol, and dermal Treg analysed 2 weeks later. **A.** *Left* - representative overlaid contour plots showing CD25 and CTLA-4 expression by $CD4^+CD25^{low\ to\ +}$ Tconv and $CD4^+CD25^{high}$ Treg. *Right* - summary box and whiskers plot showing CTLA-4 geometric mean fluorescent intensity ± range. BMT n=4, BMT + T n= 4 from one experiment. **B.** Representative histograms showing the expression of ICOS and CD103 on gated $CD4+CD25^{low\ to\ +}FoxP3^{neg}$ Tconv or $CD4+CD25^{high}FoxP3^+$ Treg. **C.** Summary box and whiskers plots showing the geometric mean fluorescent intensity ± range for ICOS and CD103 on gated T cells. BMT n=8, BMT + T n=11. Data are pooled from 2 independent experiments. All experiments were analysed using Mann Whitney tests. *P<0.05, **P<0.01, ***P<0.001.
